# Supplementary material for: Mitofusin-2 stabilizes adherens junctions and suppresses endothelial inflammation via modulation of β-catenin signaling
Source: Nat Commun. 2021 May 12;12:2736. doi: 10.1038/s41467-021-23047-6 (PMC8115264; doi:10.1038/s41467-021-23047-6)
Supplement: Supplementary file 1 — Supplementary Information [file 41467_2021_23047_MOESM1_ESM.pdf]

SUPPLEMENTARY INFORMATION

Mitofusin-2 Stabilizes Adherens Junctions and Suppresses Endothelial Inflammation via Modulation of β-catenin Signaling

Young-Mee Kim[1,2,3]*, Sarah Krantz[1,2], Ankit Jambusaria[1,2,4], Peter T. Toth[2,5], Hyung-Geun Moon[6], Isuru Gunarathna[1,4], Gye Young Park[6], Jalees Rehman[1,2,3,4]*

[1]Division of Cardiology, Department of Medicine, University of Illinois at Chicago,
[2]Department of Pharmacology and Regenerative Medicine, University of Illinois at Chicago,
[3] University of Illinois Cancer Center, University of Illinois at Chicago,
[4]Department of Bioengineering, University of Illinois at Chicago,

[5]Research Resources Center, University of Illinois at Chicago,

[6]Division of Pulmonary, Critical Care, Sleep and Allergy, Department of Medicine,

University of Illinois at Chicago, Chicago, IL

SUPPLEMENTARY METHODS

High affinity LC-MS/MS Proteomic analysis. HLMVECs were infected with lenti-GFP or lenti-GFP-Mfn2 virus for 72h and followed by immunoprecipitation using GFP-trap magnetic agarose (Chromo Tek). The pull-downed protein was run on a SDS-PAGE gel and stained with Coomassie blue staining solution to check specific binding protein and pull-downed protein amount. For affinity LC-MS/MS proteomic analysis (University of North Carolina, proteomic core), the pull-downed protein was washed 3x with 1 mL ice cold lysis buffer without protease and phosphatase inhibitors, and then washed 3x with 1 mL ice cold wash buffer (20 mM Tris-HCl, pH7.8). Three replicates of each condition were digested with trypsin on beads. The data was processed using Proteome Discoverer 2.1 and searched against a Uniprot reviewed human database. A CRAPome frequency was calculated and represents the fold change (FC) for each replicate using absolute spectral counts. Proteomic datasets are provided in the Source data file and a link is also in the Data Availability section.

siRNA Transfection. HLMVECs were transfected with 20nM siRNAs using Oligofectamine (Invitrogen, 12252011) for 4h[1]. After transfection, cells were changed to growth medium and further incubated for 48 h at 37°C before experiments.

EdU (5-ethynyl-2´-deoxyuridine)-labeled cell proliferation assay. The control and Mfn2-KD ECs were synchronized in 0% FBS EBM for 24 h. For EdU incorporation[2], the cells were treated with 1 μM EdU in 10% FBS EGM2 for 16 h, and then EdU was detected using Click-It Plus EdU Alexa Fluor 647 flow cytometry assay kit (Thermo scientific, C10635). 7AAD was used to stain DNA. The stained cells were analyzed by flow cytometry assay (LSRFortessa).

Cell apoptosis assay using FITC-Annexin V. The control and Mfn2-KD ECs were trypsinized (retaining all floating cells) and stained with FITC-Annexin V and propidium iodide (PI) by using the Annexin V: FITC assay kit (Bio-Rad, ANNEX300F). The stained cells were analyzed by flow cytometry assay (LSRFortessa). The apoptotic cells are represented by double positive cells for FITC-Annexin V and PI, and expressed as % of control[3].

Confocal microscopy: 1. F-actin structure. For F-actin structure at AJ junction[4, 5], control and Mfn2-KD ECs were infected with lenti-LifeAct-EGFP for 48 h and the images were taken in live cells using confocal microscopy (Zeiss LSM880, PlanApo 1.46NA, 63x objective). For

colocalization of VE-cadherin and F-actin, the cells were fixed with 4% PFA followed by immunostaining with VE-cadherin antibody and anti-rabbit secondary Alexa568. The protein colocalization was analyzed with plot profiles using ImageJ.

2. Mitochondrial ROS assessment. To measure mitochondrial ROS generation, ECs were incubated with 5 μM of the mitochondrial ROS dye Mito-Sox (Invitrogen, M36008) for 10 min, and fluorescence intensity in live cells was measured using confocal microscopy (Zeiss LSM880, Plan Apo 1.45NA, 63x objective)[6]. The fluorescence intensity from each sample among different groups was measured under the same setting with ImageJ. Relative fluorescence intensity was expressed as fold increase from the control.

3. Mitophagy assessment. HLMVECs infected with lenti- Mfn2 shRNA and lenti-MitoKeima virus (mitoKeima construct, a gift provided by Dr. Toren Finkel at the University of Pittsburgh)[7], and then treated with doxycycline (200 ng/mL) for 72 h. Control cells were treated with DMSO. The cells were plated in glass bottom dishes and mitophagy was measured in live cells. In the alkaline environment (pH 8.0), the shorter-wavelength (green) excitation predominates, but the Keima protein undergoes a gradual shift to the longer-wavelength (red) excitation in the acidic lysosome (pH 4.5). These allow us to determine whether Keima-tagged mitochondria are at the physiological pH of the mitochondria (pH 8.0) or the lysosome (pH 4.5). For confocal microscopy (Zeiss LSM710 BiG, Plan Apo 1.46NA, 63x objective), dual-excitation ratio imaging was carried out with two sequential excitation/ emission lasers (405 and 561 nm, 677 and 579 nm, respectively). Hoechst was used to stain nuclei. The level of mitophagy was determined by fluorescence intensity ratio of A561 nm/ A405 nm and then expressed as fold change from the control.

4. Mitochondrial morphology assessment. ECs were depleted of the relevant mediators using 20 nM of siRNA for targeting Mfn2, Mfn1, Drp1, or Opa1 with single, double, or triple combinations of each siRNA for 48h. An antibody targeting the mitochondrial membrane protein Tom20 (1:100 dil, sc-17764) was used to visualize the mitochondrial network and anti-VE-cadherin (1:250 dil, Cayman #160840) was used to demarcate the EC barrier. Secondary antibodies with 1:500 dil of Alexa Fluor 568 (A11031) and Alexa Fluor Plus 488 (A32731) were used for the primary antibodies targeting Tom20 and VE-cadherin, respectively. Cells were imaged using confocal microscopy (Zeiss LSM770, PlanApo 1.46NA, 63x objective). Full microscopy images are provided at Source Data files.

Genotyping primers. For Mfn2 fl/fl, sense 5'- TTT GGA AGT AGG CAG TCT CCA-3', anti-sense 5'- CAG GCA GCA CTG AAA AGA GA-3'. For Cdh5-Cre, sense 5'- GAT CGC TGC CAG GAT ATA

CG-3’, anti-sense 5’- AAT CGC CAT CTT CCA GCA G-3’. All primer lists are provided at Supplementary table1 of Source Data.

Subcellular Cell Fractionation. Confluent ECs were treated with TNFα for 6 h and subjected to subcellular fractionation (cytosol, membrane: plasma membrane+ mitochondrial membrane, soluble nucleus extract) using a Subcellular Protein Fractionation Kit for Cell Culture (Thermo Scientific #78840). An equal amount of protein for subcellular fractions was loaded in SDS-PAGE to evaluate their localization. The mitochondrial proteins (Mfn2, Drp1, VDAC, and CoxIV), cytosolic proteins (GAPDH), plasma membrane proteins (Na/K ATPase) and a nuclear matrix protein (p84) were assessed by Western blotting with 1:1000 dil of specific antibodies (Mfn2 (ab56889, mouse), VDAC (CST-4661, rabbit), COXIV (11242-1-AP, rabbit), Na+/K+-ATPase (sc-21712, mouse)). membrane: memb, soluble nuclear extract: NE.

Statistical analysis. Co-localization of fluorescence images by confocal microscopy was analyzed by evaluating Spearman’s correlation. The intensity levels of Mfn1 and VE-cadherin were measured using ImageJ and then the distribution of measured intensity levels was used for Spearman’s correlation analysis.

SUPPLEMENTARY FIGURES AND LEGENDS

a

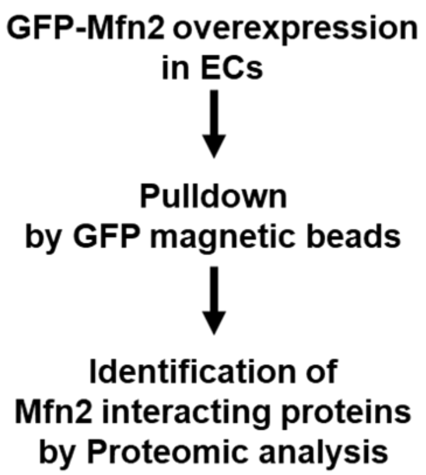

b

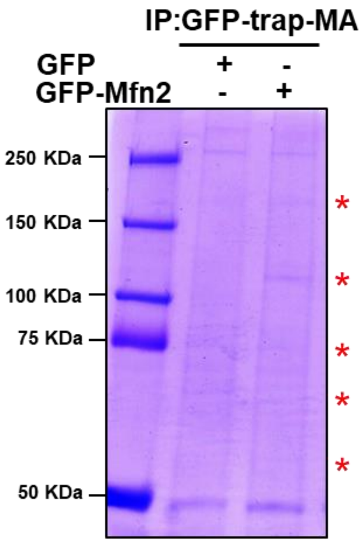

c

Mfn2 specific binding 25 proteins

KRT18, ENO1, MACF1, RPS3A, ILF2, FARSA, RPL27A, CKAP4, FAM120A, RPL32, IGF2BP3, UPF1, BAG2, MFN2, SRPRB, H2AB, PML, DYSF, SEPT9, DPM1, PRPF19, MARCH5, LMNB2

- *Filtered "Hits": Fold Change ≥ 1.5*
- *p-value using t-test, results in column AL-AN (two-tailed, homoscedastic)*
- *p-value cut-off of 0.1*
- *Must meet this criteria for at least 2 out of 3 replicates.*

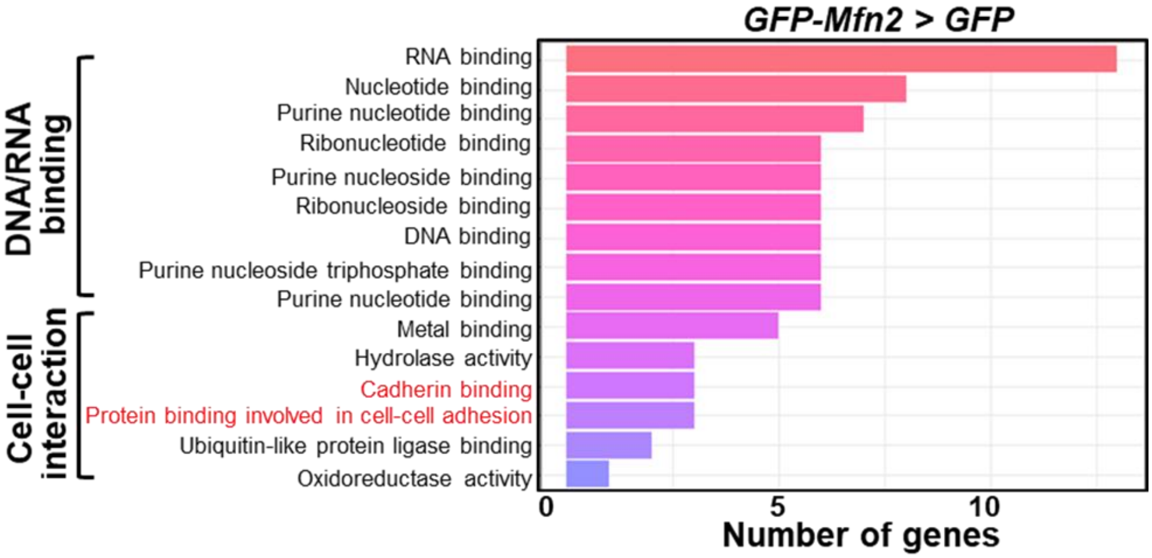

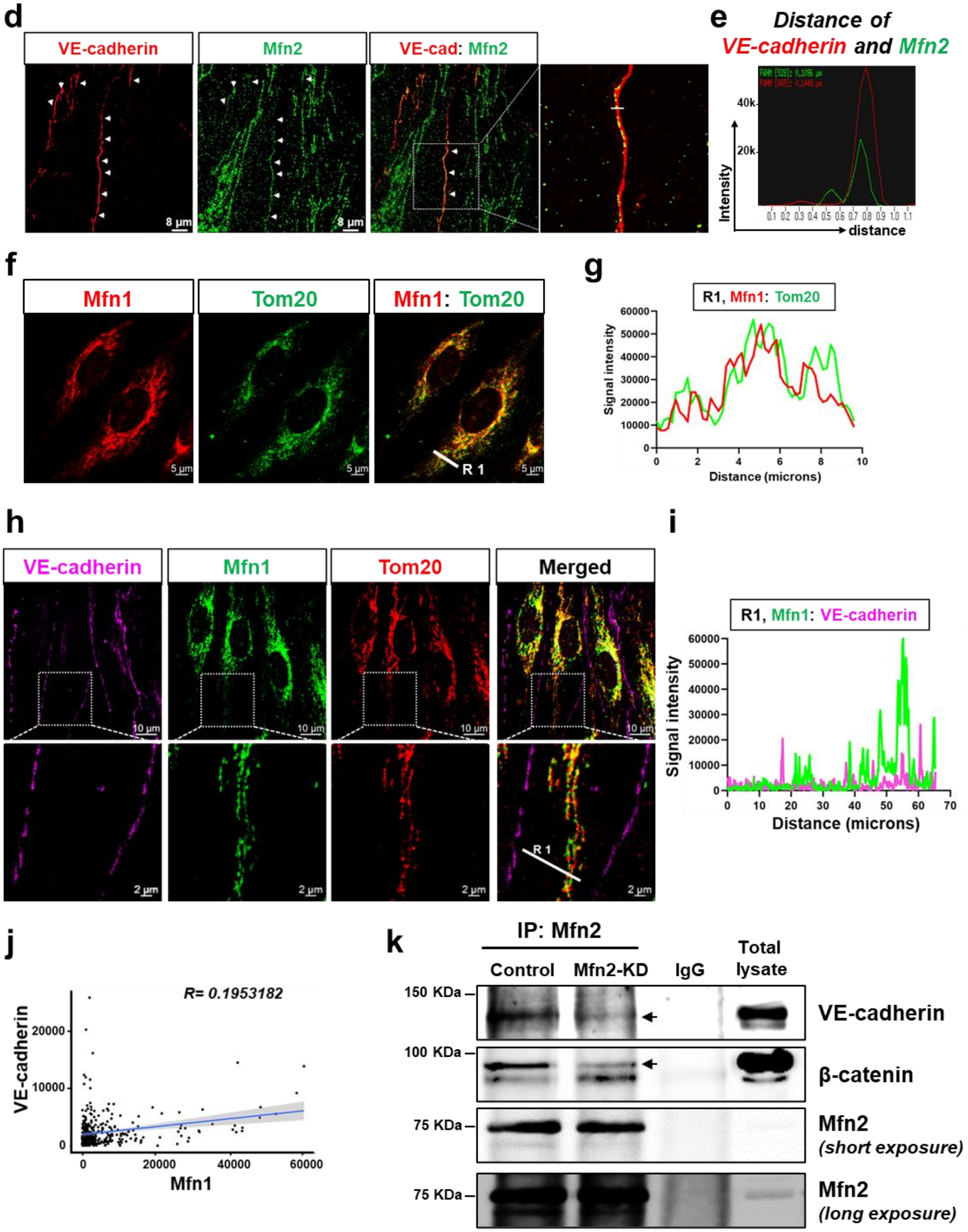

d
VE-cadherin
Mfn2
VE-cad: Mfn2
8 µm
e
Distance of VE-cadherin and Mfn2
40k
20k
Intensity
distance
f
Mfn1
Tom20
Mfn1: Tom20
5 µm
R 1
g
R1, Mfn1: Tom20
Signal intensity
Distance (microns)
h
VE-cadherin
Mfn1
Tom20
Merged
10 µm
2 µm
R 1
i
R1, Mfn1: VE-cadherin
Signal intensity
Distance (microns)
j
R= 0.1953182
VE-cadherin
Mfn1
k
IP: Mfn2
Control
Mfn2-KD
IgG
Total lysate
150 KDa
100 KDa
75 KDa
VE-cadherin
β-catenin
Mfn2 (short exposure)
Mfn2 (long exposure)

Supplementary Figure 1. Identification of Mfn2 interacting proteins in homeostatic ECs and verification of interacting Mfn2 and AJs complex. (a-b) HLMVECs were infected with lenti-GFP or lenti-GFP-Mfn2 virus for 72 h followed by immunoprecipitation using GFP-trap magnetic beads. The immunoprecipitated proteins were run on an SDS-PAGE gel **(b)** and stained with Coomassie blue staining solution to check specific binding and protein amount, then underwent affinity LC-MS/MS proteomic analysis. Three replicates of each condition showed similar patterns on Coomassie blue stained SDS-PAGE gels. **(c)** ***Upper panel,*** the list of identified Mfn2-binding partner proteins in ECs. Three replicates of each condition were digested with trypsin on beads. The data was processed using Proteome Discoverer 2.1 and analyzed using the UNIPROT database. A CRAPome frequency was calculated and represents the fold change (FC) for each replicate using absolute spectral counts (see Source data and Data Availability sections). ***Lower panel,*** gene ontology enrichment analysis. All data were used to categorize the function of Mfn2 partners. **(d-e)** Confluent HLMVECs were fixed with 100% methanol for 5 min on -20 ºC and permeabilized with 0.25% Triton X-100 for 10 min. **(d)** Mfn2 cellular localization was examined by Mfn2 immunostaining with co-staining of the AJ protein VE-cadherin. The images were taken using 3D-SIM (DeltaVision OMX in 63x magnification). The Z-axis sections (28-30 sections, 125nm step size) were taken at full-frame structured illumination mode (1024x1024 pixel, sequential acquisition). Softworx (Applied Precision) was used to reconstruct 3D-SIM images and the projection images are presented. Mfn2 and VE-cadherin were pseudocolored with green and red, respectively. The white arrow heads represent the colocalized Mfn2 at the AJs VE-cadherin. The area with white box was enlarged in the right panel and the yellow color indicates colocalization of Mfn2 and VE-cadherin. **(e)** The white line in **(d)** indicate the area where the distance was analyzed between Mfn2 and VE-cadherin using Softworx7.0.0. **(f-j)** Confluent HLMVECs were fixed with 4% PFA for 10 min at RT and permeabilized with 0.25% Triton X-100 for 10 min. Mfn1 cellular localization was examined by Mfn1 immunostaining with co-staining of Tom20 **(f)** or VE-cadherin and Tom20 **(h)**. The images were taken using confocal microscopy (Zeiss LSM770, Plan Apo 1.46NA, 63x objective). We evaluated colocalization of Mfn1 and Tom20 or VE-cadherin, Mfn1, and Tom20 in more than three independent experiments using confocal microscopy and observed results similar to those shown in the representative images. **(g and i)** R1 of **f** and **h** was used for analyzing colocalization of Mfn1 and Tom20, or Mfn1 and VE-cadherin, respectively. **(j)** Spearman's correlation analysis between Mfn1 and VE-cadherin in **(h)**. The plot additionally contains a best fit line (blue) with a 95% confidence interval. **(k)** HLMVECs were infected with doxycycline inducible lenti-Mfn2 shRNA virus for 48 h and treated with doxycycline for 72h to deplete endogenous Mfn2 (Mfn2-KD). DMSO was used as a vehicle for control ECs.

Mfn2 were immunoprecipitated with specific antibody followed by Western blotting for VE-cadherin or β-catenin antibodies. Mouse normal IgG was used for negative control in immunoprecipitation assay. The representative images were chosen from n=4 independent biological experiments which showed consistent results. Uncropped blots can be found in the Source Data file.

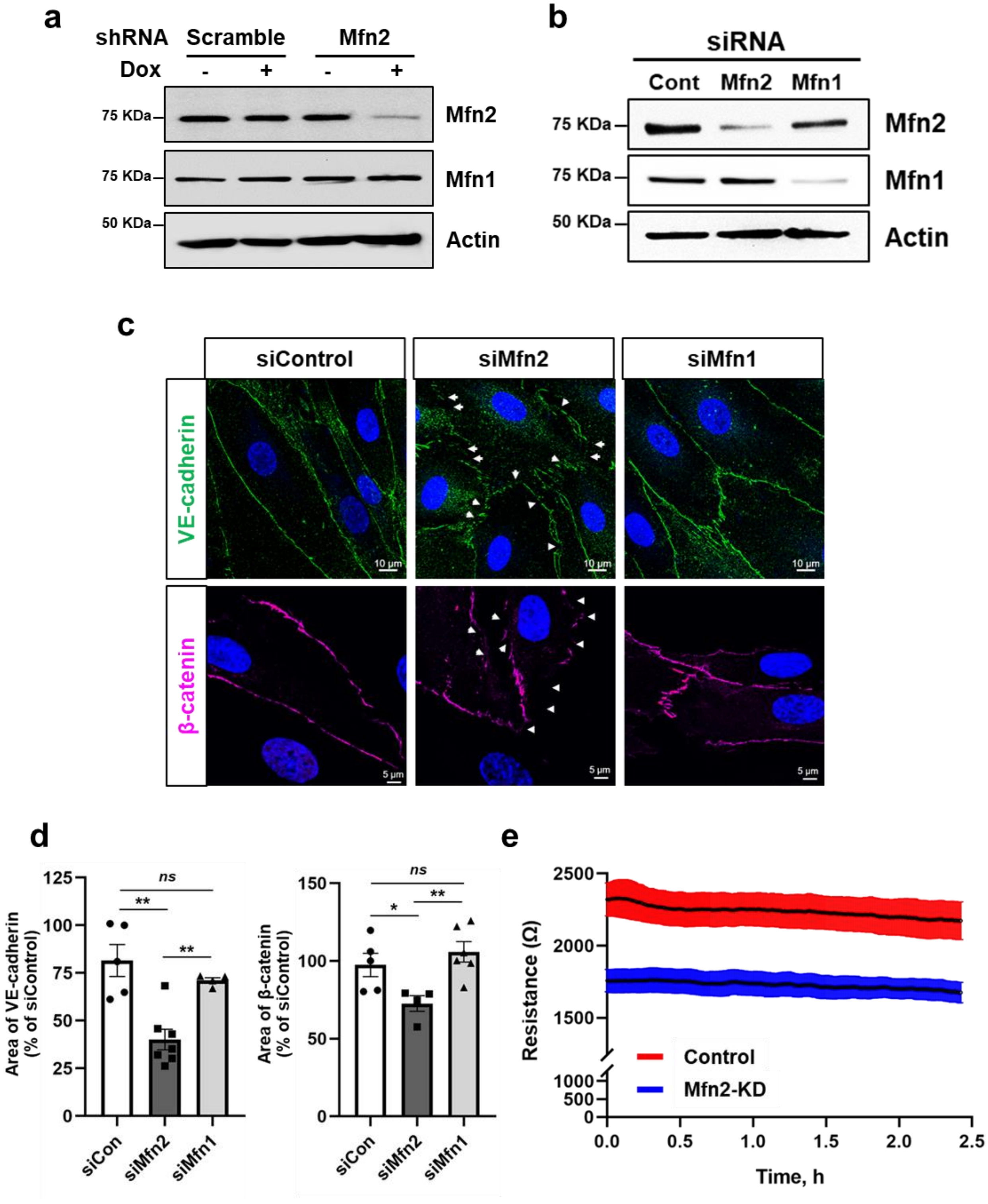

a
shRNA
Scramble
Mfn2
Dox
- + - +
75 KDa
75 KDa
50 KDa
Mfn2
Mfn1
Actin
b
siRNA
Cont
Mfn2
Mfn1
75 KDa
75 KDa
50 KDa
Mfn2
Mfn1
Actin
c
siControl
siMfn2
siMfn1
VE-cadherin
β-catenin
10 µm
5 µm
d
Area of VE-cadherin (% of siControl)
Area of β-catenin (% of siControl)
ns
**
*
siCon
siMfn2
siMfn1
e
Resistance (Ω)
Control
Mfn2-KD
Time, h

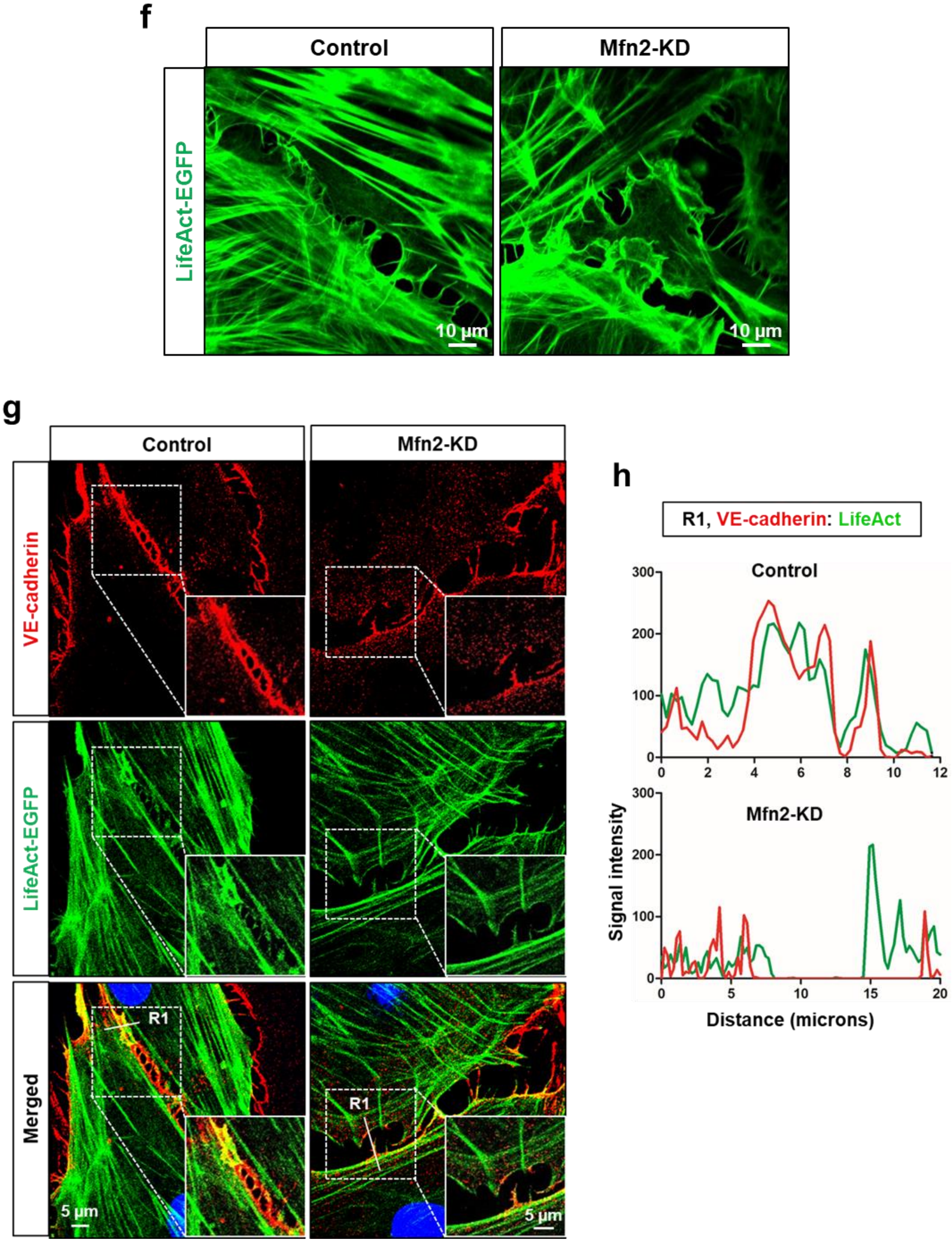

Supplementary Figure 2. Mfn2 specifically stabilizes AJ junctions supported by F-actin in

homeostatic EC. (a) HLMVECs were infected with lenti-shRNA (scramble control or Mfn2) and treated with doxycycline for 72 h. Mfn2 knock-down efficiency was measured by Western blotting. We confirmed similar Mfn2 knock-down efficiency in at least give independent experiments). Uncropped blots can be found in the Source Data file. **(b-d)** HLMVECs were transfected with siRNA for scramble control, Mfn2, or Mfn1 for 48h. **(b)** The knock-down efficiency of Mfn2 or Mfn1 was measured by Western blotting with their specific antibodies. We obtained similar results in at least three independent experiments. Uncropped blots can be found in the Source Data file. **(c)** Barrier integrity of Mfn2 or Mfn1 depleted ECs with siRNA was examined by VE-cadherin and β-catenin immunostaining using confocal microscopy (Zeiss LSM770, Plan Apo 1.46NA, 63x objective). **(d)** The levels of VE-cadherin and β-catenin at the cell surface in **c** were determined by analyzing their area using ImageJ and are represented as % of control si-RNA (siControl). Data are mean values ± SEM from n= 4-7 independent biological samples. ***p=0.0014* for the area of VE-cadherin in control siRNA vs siMfn2, ***p=0.0023* for the area of VE-cadherin in siMfn2 vs siMfn1, **p=0.0367 for* area of β-catenin in control siRNA vs siMfn2, ***p=0.0065 for* area of β-catenin in siMfn2 vs siMfn1 by unpaired, two-tailed t-test, ns; no significant. **(e)** HLMVECs were infected with lenti Mfn2 shRNA virus and treated with doxycycline for 72 h. Control cells were treated with DMSO. ECs barrier resistance was measured in control and Mfn2-KD ECs with TER. The data are presented without normalizing to show the baseline values. Data are mean values ± SEM for 5 independent experiments. **(f)** F-actin structure of control and Mfn2-KD ECs was examined by overexpressing LifeAct-EGFP in live cells using confocal microscopy (Zeiss LSM880, PlanApo 1.46NA, 63x objective). The representative images were from 3 independent experiments which were showed similar results. **(g)** Correlation of VE-cadherin and F-actin of control and Mfn2-KD ECs was examined by overexpressing LifeAct-EGFP and immunostaining with VE-cadherin using confocal microscopy (Zeiss LSM880, PlanApo 1.46NA, 63x objective). **(h)** The white line, R1 at **g** indicates the area analyzing the distance between F-actin (LifeAct) and VE-cadherin using ImageJ. The images are representative of at least 3 independent experiments.

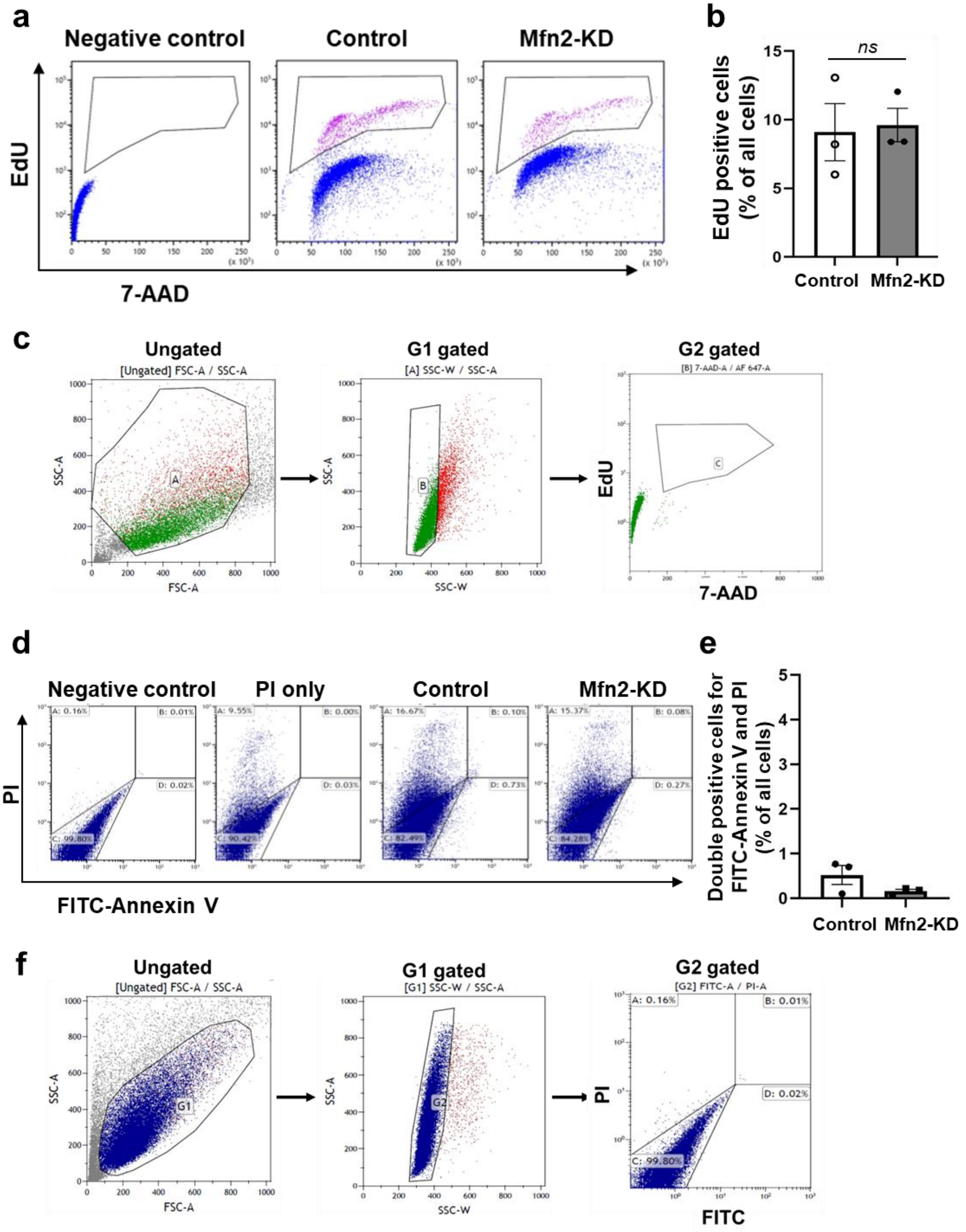
a
Negative control
Control
Mfn2-KD
EdU
7-AAD
b
EdU positive cells
(% of all cells)
ns
Control
Mfn2-KD
c
Ungated
[Ungated] FSC-A / SSC-A
SSC-A
FSC-A
A
G1 gated
[A] SSC-W / SSC-A
SSC-W
B
G2 gated
[B] 7-AAD-A / AF 647-A
C
EdU
7-AAD
d
Negative control
PI only
Control
Mfn2-KD
A: 0.16%
B: 0.01%
C: 99.80%
D: 0.02%
A: 9.55%
B: 0.00%
C: 90.42%
D: 0.03%
A: 16.67%
B: 0.10%
C: 82.49%
D: 0.73%
A: 15.37%
B: 0.08%
C: 84.28%
D: 0.27%
PI
FITC-Annexin V
e
Double positive cells for
FITC-Annexin V and PI
(% of all cells)
Control
Mfn2-KD
f
Ungated
[Ungated] FSC-A / SSC-A
SSC-A
FSC-A
G1
G1 gated
[G1] SSC-W / SSC-A
SSC-W
G2
G2 gated
[G2] FITC-A / PI-A
A: 0.16%
B: 0.01%
C: 99.80%
D: 0.02%
PI
FITC

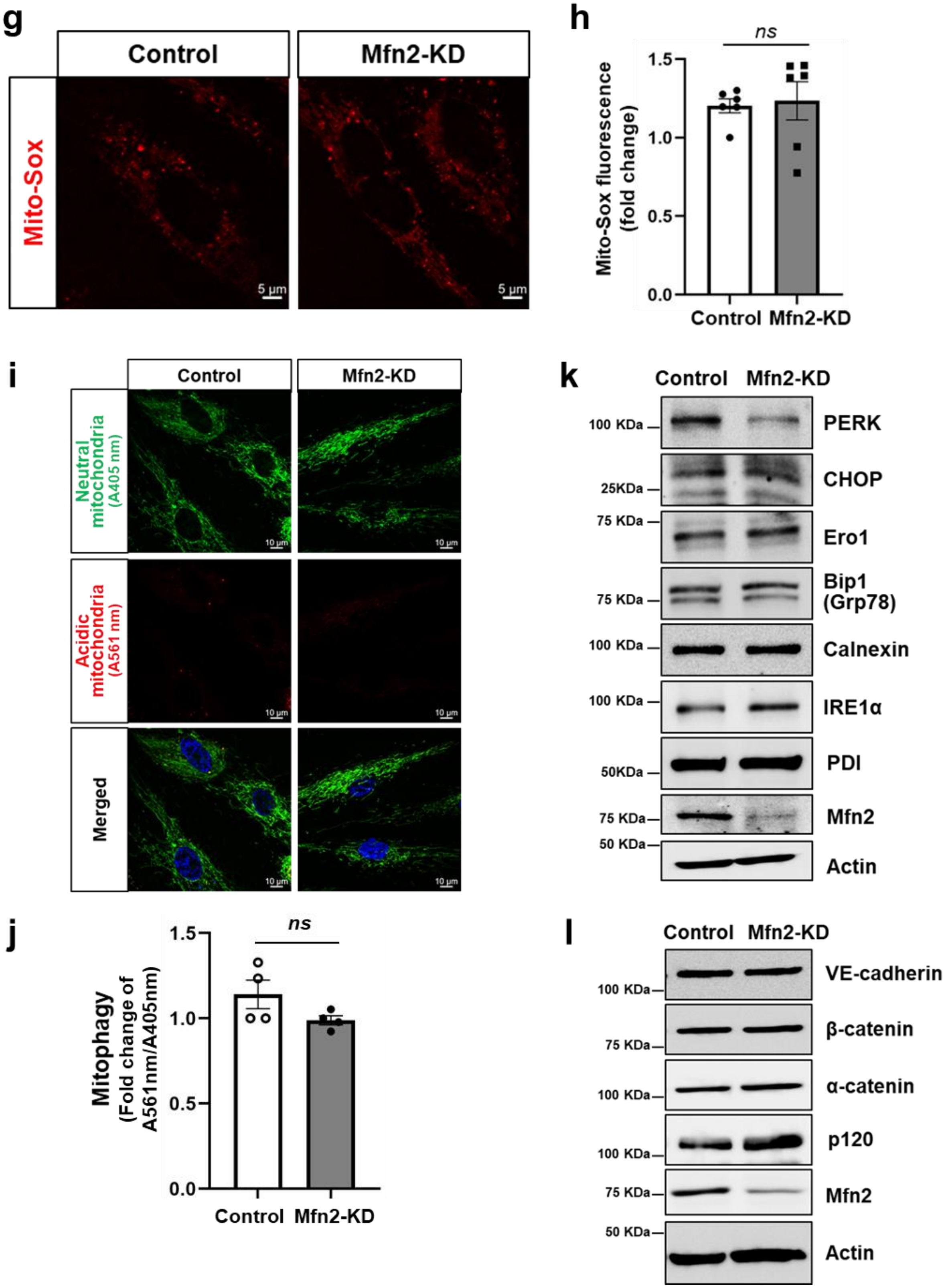
g
Control
Mfn2-KD
Mito-Sox
5 µm
5 µm
h
ns
Mito-Sox fluorescence (fold change)
1.5
1.0
0.5
0.0
Control
Mfn2-KD
i
Control
Mfn2-KD
Neutral mitochondria (A405 nm)
Acidic mitochondria (A561 nm)
Merged
10 µm
k
Control
Mfn2-KD
100 KDa
PERK
25KDa
CHOP
75 KDa
Ero1
75 KDa
Bip1 (Grp78)
100 KDa
Calnexin
100 KDa
IRE1α
50KDa
PDI
75 KDa
Mfn2
50 KDa
Actin
j
ns
Mitophagy (Fold change of A561nm/A405nm)
1.5
1.0
0.5
0.0
Control
Mfn2-KD
l
Control
Mfn2-KD
100 KDa
VE-cadherin
75 KDa
β-catenin
100 KDa
α-catenin
100 KDa
p120
75 KDa
Mfn2
50 KDa
Actin

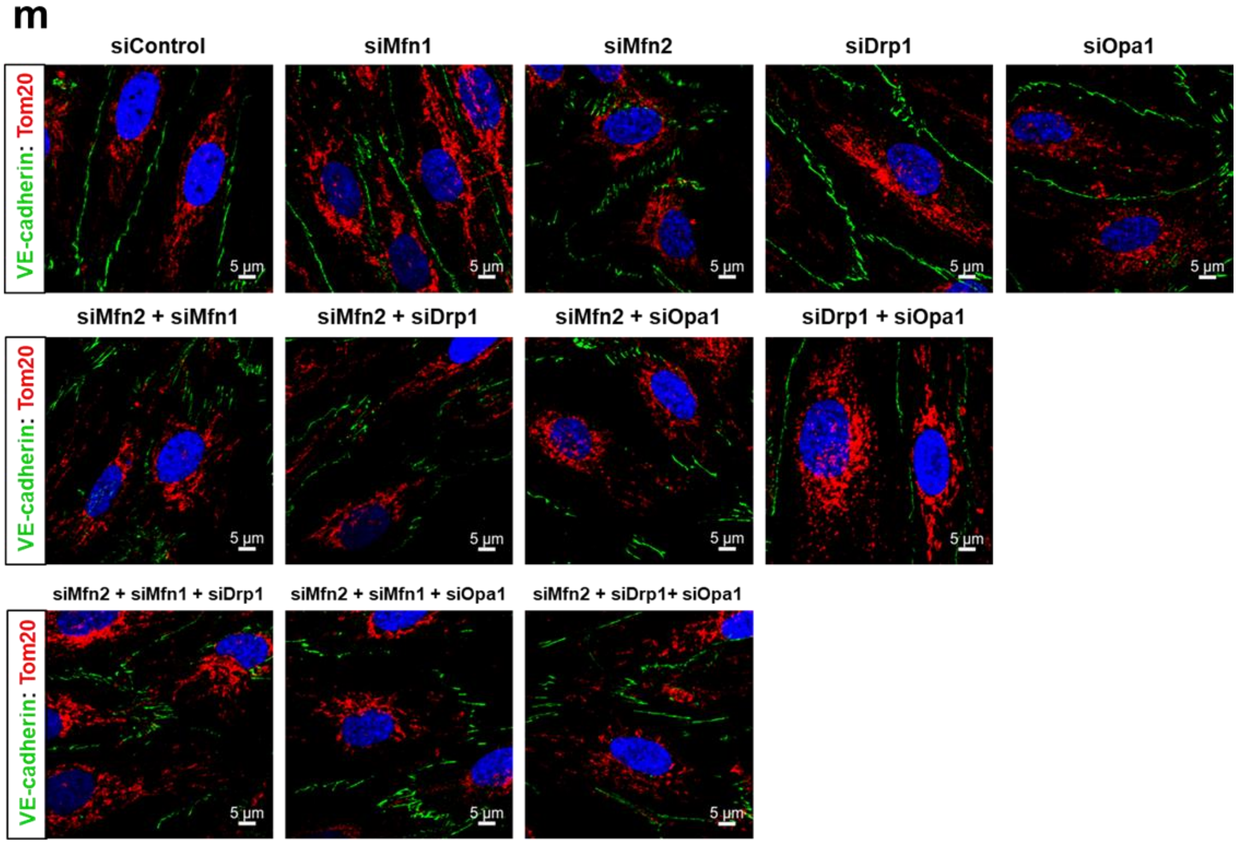

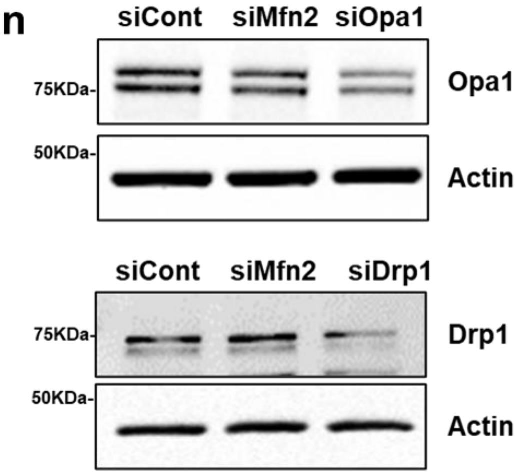

Supplementary Figure 3. Mfn2 depletion did not impact general stresses and AJs protein levels. HLMVECs were infected with lenti Mfn2 shRNA virus and treated with doxycycline for 72 h. Control cells were treated with DMSO. **(a-c)** The cells were stained for EdU to evaluate proliferation and analyzed by FACS. 7-AAD was used to stain DNA. **(a)** The dot plots from FACS analysis show EdU and 7-AAD fluorescence. **(b)** The bar graph was presented as % of EdU positive cells in total cells. Data are presented from 3 independent experiments. ***ns***: no

significance, *p=0.8837* by paired, two-tailed t-test. **(c)** FACS sequential gating strategies were presented to evaluate EdU positive cells for **a** and **b**. **(d-f)** The cells were stained with FITC-Annexin V to evaluate apoptosis and analyzed by FACS. Propidium Iodide (PI) was used to stain DNA. **(d)** The dot plots from FACS analysis were presented with FITC-Annexin V and PI. **(e)** The bar graph was presented as % of double positive cells of FITC-Annexin V and PI in total cells. Data are mean values ± SEM for 3 independent experiments. ***ns***: no significance. **(f)** FACS sequential gating strategies are presented to evaluate apoptotic cells for **d** and **e**. **(g-h)** The cells were evaluated for mitochondrial ROS production with Mito-Sox staining and images were obtained using confocal microscopy (Zeiss LSM770, PlanApo 1.46NA, 63x objective). **(g)** The representative images for Mito-Sox were chosen from n=6 independent biological replicates. **(h)** The relative Mito-Sox fluorescence intensity of **g** was presented as fold change. Data are mean values ± SEM for n=6 independent biological replicates. ***ns***: no significance, *p=0.8268* by paired, two-tailed t-test. **(i-j)** The control and Mfn2-KD ECs were transfected with the mito-Keima construct and evaluated for mitophagy. **(i)** The representative images for mito-Keima were chosen from n=4 independent experiments. Neutral mitochondria are shown in green color at A405 nm and acidic mitochondria by mitophagy are shown in red color at A561 nm. **(j)** The quantification of mitophagy was presented by fold change of acidic mitochondria by normalizing against neutral mitochondria. Data are presented from 4 independent experiments. ***ns***: no significance, *p=0.1092* by paired, two-tailed t-test. **(k)** ER stress by Western blotting with specific ER stress markers. The images are representative of at least 3 independent experiments. Uncropped blots can be found in the Source Data file. **(l)** Protein expression levels of AJ complexes (VE-cadherin, β-catenin, α-catenin, and p120) were measured by Western blotting. The images are representative of at least 3 independent experiments. Uncropped blots can be found in the Source Data file. **(m)** ECs were depleted of mitochondrial dynamics mediators using specific siRNA for Mfn2, Mfn1, Drp1, or Opa1 with single, double, or triple combination of each siRNA for 48h. Tom20 (red) was used to visualize the mitochondrial network and VE-cadherin (green) was used to demarcate the EC barrier. Cells were imaged using confocal microscopy (Zeiss LSM770, PlanApo 1.46NA, 63x objective). The representative images were selected from 3 independent experiments which showed similar results. **(n)** The knock-down efficiency of Opa1 and Drp1 was determined by Western blotting with antibodies against Opa1 (1:1000 dil, CST-80471) and Drp1 (1:1000 dil, sc-271583). Uncropped blots can be found in the Source Data file. The representative western blots were selected from 3 independent experiments which showed similar results.

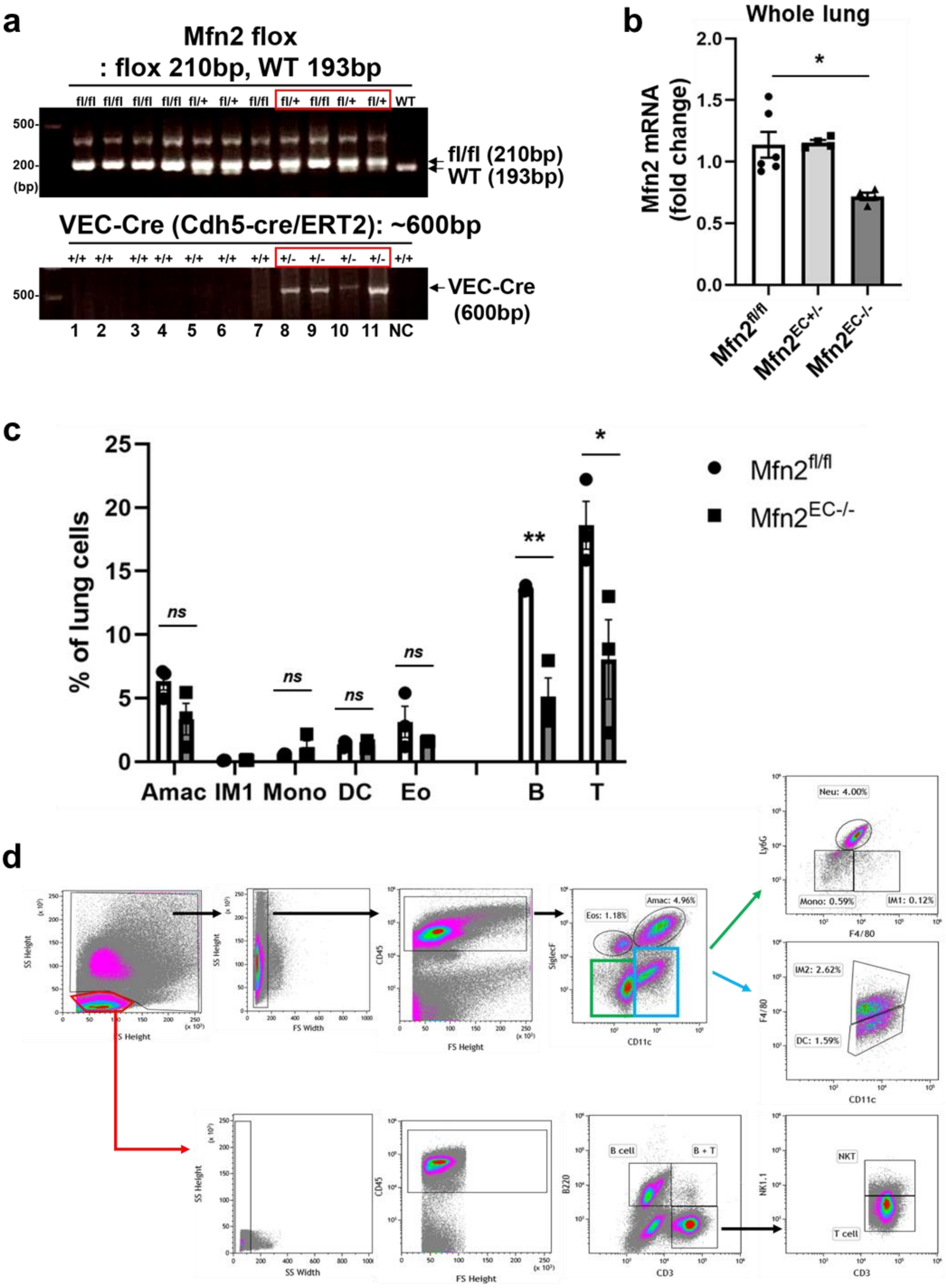

a
Mfn2 flox
: flox 210bp, WT 193bp
fl/fl (210bp)
WT (193bp)
VEC-Cre (Cdh5-cre/ERT2): ~600bp
VEC-Cre (600bp)
1 2 3 4 5 6 7 8 9 10 11 NC
b
Whole lung
Mfn2 mRNA (fold change)
Mfn2fl/fl
Mfn2EC+/-
Mfn2EC-/-
c
% of lung cells
Amac IM1 Mono DC Eo B T
Mfn2fl/fl
Mfn2EC-/-
d
Neu: 4.00%
Mono: 0.59%
IM1: 0.12%
Eos: 1.18%
Amac: 4.96%
IM2: 2.62%
DC: 1.59%
B cell
B + T
NKT
T cell
SS Height
FS Height
FS Width
CD45
SiglecF
CD11c
Ly6G
F4/80
SS Width
B220
CD3
NK1.1

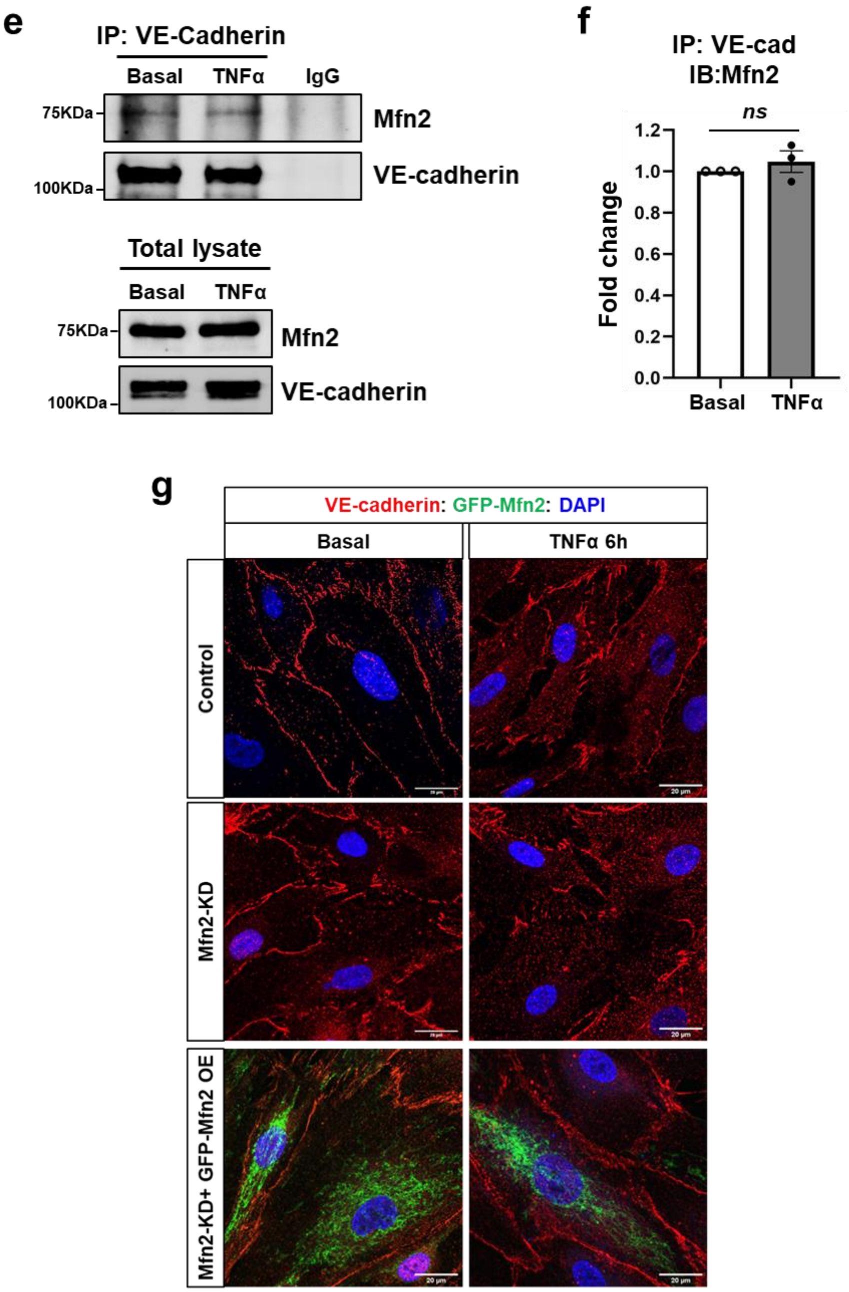

Supplementary Figure 4. Characterization of endothelial specific conditional Mfn2 knockout mice and rescue effect of GFP-Mfn2 in TNFα induced AJs disassembly. (a) *Generation of endothelial specific conditional Mfn2 knockout mice*: Mfn2 flox mice were bred with tamoxifen inducible VE-Cadherin-Cre (Cdh5)-ERT2 transgenic mice to make EC specific conditional Mfn2 knockout mice. The genotyping results in agarose gel show Mfn2 fl/fl (or fl/+) and Mfn2$^{EC-/-}$ (Mfn2 fl/fl:VEC-Cre+) mice. All mice were genotyped before tamoxifen administration. **(b)** All mice received tamoxifen and were rested for one month before

experiments. The lungs from control (Mfn2fl/fl, n=6), Mfn2$^{EC+/-}$(Mfn2fl/+, Cdh5-cre/ERT2, n=4), or Mfn2$^{EC-/-}$(Mfn2fl/fl, Cdh5-cre/ERT2, n=3) mice, were excised and Mfn2 knockout efficiency was evaluated by RT-qPCR. 18S was used a control and Mfn2 mRNA levels were normalized by 18S levels and represented by fold change. Data are mean values ± SEM for n=3-6 mice. **p=0.0135* for Mfn2$^{EC-/-}$ *vs* Mfn2$^{fl/fl}$ by unpaired, two-tail t-test. **(c)** Lungs from Mfn2$^{fl/fl}$ (littermates of Mfn2$^{EC-/-}$ mice) and Mfn2$^{EC-/-}$ mice were homogenized and inflammatory cells were stained with indicated specific antibodies by following FACS analysis. The inflammatory cells are represented as percent (%) of whole lung cells. Data are mean values ± SEM for n=3 mice. **Amac**: alveolar macrophage, **IM1**: Interstitial macrophages 1, **Mono**: monocyte, **B**: B cells, **T**: T cells, **Eo**: Eosinophil. **p=0.0445*, ***p=0.0043* for Mfn2$^{EC-/-}$ vs Mfn2$^{fl/fl}$ by unpaired, two-tailed t-test, ***ns***: no significant. **(d)** FACS sequential gating strategies for **c** and main figures 3g-h. **(e)** Confluent HLMVECs were treated with TNFα (10 ng/mL) for 6 h. VE-cadherin was immunoprecipitated with VE-cadherin specific antibody followed by Western blotting for VE-cadherin or Mfn2 antibodies. Uncropped blots can be found in the Source Data file. **(f)** The band intensities for Mfn2 and VE-cadherin interactions in **e** were quantified with ImageJ and are presented as fold change. Data are presented were obtained from 3 independent experiments. ***ns***: no significance, *p=0.4560* by paired, two-tailed t-test. **(g)** Rescue effect of GFP-Mfn2 on Mfn2 depletion induced EC barrier disruption: Barrier integrity of confluent control, Mfn2-KD ECs, and Mfn2-KD+ GFP-Mfn2 OE ECs were treated with or without TNFα (10 ng/mL) for 6 h and examined by VE-cadherin immunostaining using confocal microscopy (Zeiss LSM770, PlanApo 1.46NA, 63x objective). GFP-Mfn2 overexpressing cells show green. Scale bars are 20 μm. The representative images were selected from 3 independent experiments which showed similar results.

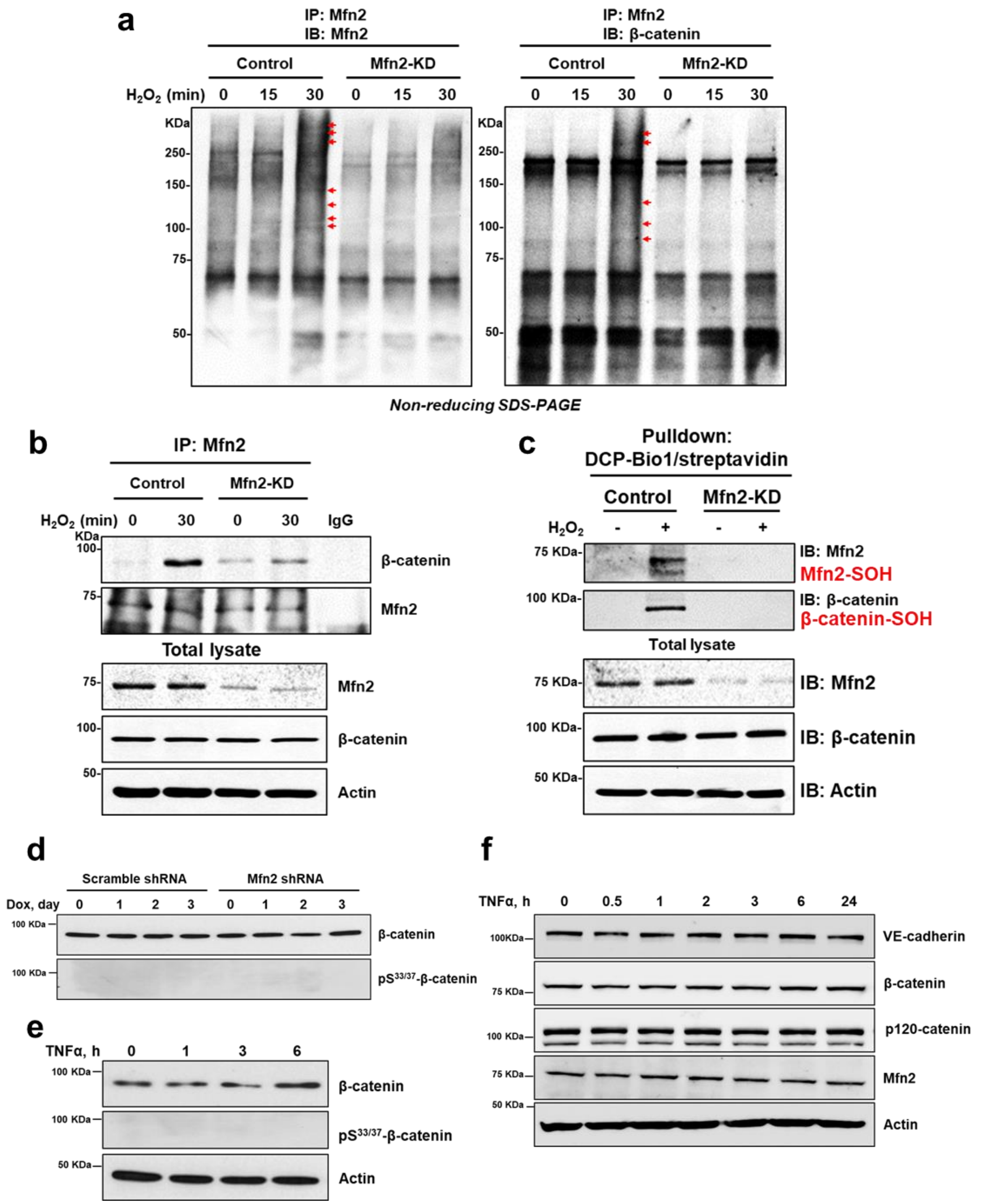

Supplementary Figure 5. Mfn2 interaction with β-catenin depends on the redox status. (a-b) Confluent HLMVECs were stimulated with H_2O_2 (500 μM) for indicated times (0, 15, or 30 min)

and immunoprecipitated with Mfn2 antibody followed by Western blotting with Mfn2 and β-catenin antibodies under non-reducing SDS-PAGE (without β-mercaptoethanol) **(a)** and reducing SDS-PAGE **(b)**. Red arrow heads indicate the shifted bands by complexes of Mfn2 or β-catenin. Mouse normal IgG was used as a negative control for immunoprecipitation. The representative images were selected from 3 independent experiments which showed similar results. Uncropped blots can be found in the Source Data file. **(c)** Control and Mfn2 depleted (Mfn2-KD) ECs were treated with 500 µM H_2O_2 for 30 min and subjected to the DCP-Bio1 assay. The total sulfenylated proteins were pulled down with streptavidin agarose beads and the sulfenylation of Mfn2 and β-catenin was determined by Western blotting. The representative images were presented from 3 independent experiments which showed similar results. Uncropped blots can be found in the Source Data file. **(d)** HLMVECs were infected with lenti-shRNA (scramble control or Mfn2) and treated with doxycycline for indicated times (0, 1, 2, or 3 days). Control cells (0 days) were treated with DMSO for 1 day. The levels of β-catenin and phospho-$S^{33,37}$-β-catenin (1:500 dil, CST-9561) were examined by Western blotting. Uncropped blots can be found in the Source Data file. **(e)** HLMVECs were stimulated with TNFα (10 ng/mL) for the indicated times (0, 1, 3, or 6 h) followed by Western blotting with antibodies for β-catenin or phospho-$S^{33,37}$-β-catenin. Uncropped blots can be found in the Source Data file. **(f)** HLMVECs were stimulated with TNFα (10 ng/mL) for the indicated times (0, 0.5, 1, 2, 3, 6, or 24 h) followed by Western blotting with specific antibodies for VE-cadherin, β-catenin, or p120-catenin. Uncropped blots can be found in the Source Data file. The representative images of **d**, **e**, or **f** were selected from 3 independent experiments which showed similar results.

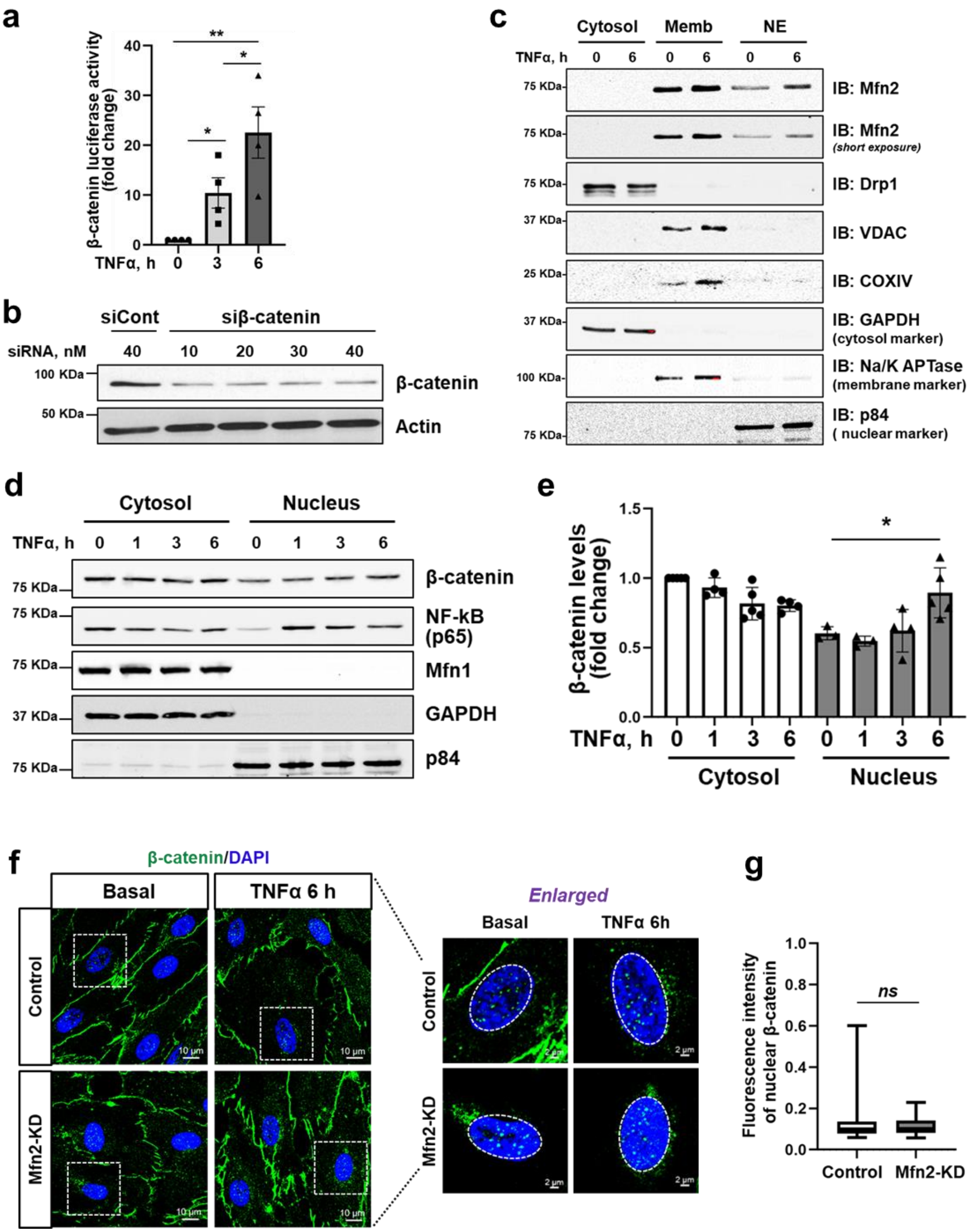

Supplementary Figure 6. TNFα increases β-catenin transcriptional activity. **(a)** HLMVECs were transfected with 1 µg Topflash (β-catenin reporter) and 35 ng of pRL/TK for 48h and

stimulated with TNFα (10 ng/mL) for indicated times (0, 3, or 6 h). Firefly and renilla luciferase activities were determined by the dual luciferase reagent assay system (Promega). Transcriptional activity of β-catenin was presented as fold change by normalizing the values versus baseline values (TNFα 0 h). Data are mean values ± SEM for n=4 independent experiments. *$p=0.0212$ for TNFα 0 h vs 3 h, *$p=0.0132$ for TNFα 3 h vs 6 h **$p=0.0057$ *for* TNFα 0 h vs 6 h by unpaired or paired, two-tailed t-test. **(b)** β-catenin knock-down efficiency by siRNA transfection in ECs. The representative images were selected from 3 independent experiments which showed similar results. Uncropped blots can be found in the Source Data file. **(c)** Confluent ECs were treated with TNFα for 6 h and subjected to subcellular fractionation (cytosol, membrane: plasma membrane+ mitochondrial membrane, soluble nucleus extract). An equal amount of protein for subcellular fractions was loaded in SDS-PAGE to evaluate their localization. The mitochondrial proteins (Mfn2, Drp1, VDAC, and COX IV), cytosolic proteins (GAPDH), plasma membrane (Na/K ATPase) and nuclear matrix protein (p84) were determined by Western blotting. membrane: memb, soluble nuclear extract: NE. The representative images were presented from 3 independent experiments which showed similar results. Uncropped blots can be found in the Source Data file. **(d-e)** HLMVECs were stimulated with TNFα (10 ng/mL) for the indicated times (0, 1, 3, or 6 h) and the cells were used for subcellular fractionation assay. **(d)** The same amount of cytosolic and nuclear fraction was loaded in the SDS-PAGE gel followed by Western blotting with specific antibodies. NF-κB (p65) was used as a positive control for nuclear translocation by TNFα. Mfn1 was used a negative control. GAPDH and p84 were used as controls for the cytosolic or nuclear fraction, respectively. The images are representative of at least 3 independent experiments. Uncropped blots in Source Data. **(e)** Quantification of β-catenin protein levels in **c**. The Western blot band intensity was quantified with ImageJ. Data are mean values ± SEM for 3-5 independent experiments. *$p=0.0185$ for TNFα 0 h *vs* 6 h by unpaired, one-tailed t-test. **(f)** Control and Mfn2-KD ECs were stimulated with TNFα (10 ng/mL) and β-catenin cellular localization in the nucleus was examined by β-catenin immunostaining and the images were taken using confocal microscopy (Zeiss LSM880, Plan Apo 1.46NA, 63x objective). Nuclei were stained with DAPI. **(g)** Quantification of nuclear β-catenin in **f**. Data are presented with box and whiskers plot and whiskers are Min to Max. Control (n=42), Mfn2-KD (n=45). Between group comparison was performed using the non-parametric Mann-Whitney test because the data were not normally distributed. ***ns***: no significant. $p=0.2796$ by paired, two-tailed t-test.

SUPPLEMENTARY TABLE

**Human gene**	qPCR primer sequences
Mfn2	sense 5'- CATCCCCAGTTGTCCTCAAG -3': anti-sense 5'-CAAGCCGTCTATCATGTCCTG-3'
ICAM-1	sense 5'-CGTGCCGCACTGAACTGGAC-3': anti-sense 5'-CCTCACACTTCACTGTCACCT-3'
IL-18	sense 5'-CAGACCTTCCAGATCGCTTC-3': anti-sense 5' -GGGTGCATTATCTCTACAGTCAGAA-3'
IL-1β	sense 5'-CCAGGGACAGGATATGGAGCA-3': anti-sense 5'- TTCAACACGCAGGACAGGTACAG-3'
IL-6,	sense 5'-TAGCCGCCCCACACAGACAG-3': anti-sense 5'-GGCTGGCATTTGTGGTTGGG-3'
B2M	sense 5′-GGTTTCATCCATCCGACATT-3′: anti-sense 5′- ATCTTTTTCAGTGGGGGTGA-3′
**Mouse genes**	qPCR primer sequences
Mfn2	sense 5'-CAAGACCGGCTGAGGTTTATT-3': anti-sense 5′-CCTTTCCACTTCCTCCGTAATC-3'
IL-1β	sense 5'-CCTTCCAGGATGAGGACATGA-3': anti-sense 5'-TGAGTCACAGAGGATGGGCTC-3'
IL-6	sense 5'-CTTCCATCCAGTTGCCTTCTTG-3': anti-sense 5'- AATTAAGCCTCCGACTTGTGAAG-3'
TNFα	sense 5'-ACGGCATGGATCTCAAAGAC-3': anti-sense 5'- AGATAGCAAATCGGCTGACG-3'
IFNγ	sense 5'-ACAATGAACGCTACACACTGCAT-3': anti-sense 5'-TGGCAGTAACAGCCAGAAACA-3'
**Mouse genes**	Genotyping primer sequences
Mfn2 fl/fl	sense 5′-TTTGGAAGTAGGCAGTCTCCA-3′: anti-sense 5′-CAGGCAGCACTGAAAAGAGA-3′
Cdh5-Cre	sense 5′-GATCGCTGCCAGGATATACG-3′: anti-sense 5′-AATCGCCATCTTCCAGCAG-3′

SUPPLEMENTARY REFERENCES

1. Kim, Y.M. *et al.* ROS-induced ROS release orchestrated by Nox4, Nox2, and mitochondria in VEGF signaling and angiogenesis. *Am J Physiol Cell Physiol* **312**, C749-C764 (2017).
2. Zhu, H.E., Yin, J.Y., Chen, D.X., He, S. & Chen, H. Agmatinase promotes the lung adenocarcinoma tumorigenesis by activating the NO-MAPKs-PI3K/Akt pathway. *Cell Death Dis* **10**, 854 (2019).
3. Kim, E. *et al.* Promotion of growth factor signaling as a critical function of beta-catenin during HCC progression. *Nat Commun* **10**, 1909 (2019).
4. van Vliet, A.R. *et al.* The ER Stress Sensor PERK Coordinates ER-Plasma Membrane Contact Site Formation through Interaction with Filamin-A and F-Actin Remodeling. *Mol Cell* **65**, 885-899 e886 (2017).
5. Phng, L.K., Stanchi, F. & Gerhardt, H. Filopodia are dispensable for endothelial tip cell guidance. *Development* **140**, 4031-4040 (2013).
6. Kim, Y.M. *et al.* Redox Regulation of Mitochondrial Fission Protein Drp1 by Protein Disulfide Isomerase Limits Endothelial Senescence. *Cell Rep* **23**, 3565-3578 (2018).
7. Sun, N. *et al.* A fluorescence-based imaging method to measure in vitro and in vivo mitophagy using mt-Keima. *Nat Protoc* **12**, 1576-1587 (2017).
