# Supplementary material for: Mitofusin-2 stabilizes adherens junctions and suppresses endothelial inflammation via modulation of β-catenin signaling
Source: Nat Commun. 2021 May 12;12:2736. doi: 10.1038/s41467-021-23047-6 (PMC8115264; doi:10.1038/s41467-021-23047-6)
Supplement: Supplementary file 3 — Reporting Summary [file 41467_2021_23047_MOESM3_ESM.pdf]

nature research

Corresponding author(s): Young-Mee Kim, Jalees Rehman

Last updated by author(s): Mar 10, 2021

Reporting Summary

Nature Research wishes to improve the reproducibility of the work that we publish. This form provides structure for consistency and transparency in reporting. For further information on Nature Research policies, see our Editorial Policies and the Editorial Policy Checklist.

Statistics

For all statistical analyses, confirm that the following items are present in the figure legend, table legend, main text, or Methods section.

n/a	Confirmed	
☐	☒	The exact sample size (n) for each experimental group/condition, given as a discrete number and unit of measurement
☐	☒	A statement on whether measurements were taken from distinct samples or whether the same sample was measured repeatedly
☐	☒	The statistical test(s) used AND whether they are one- or two-sided<br>*Only common tests should be described solely by name; describe more complex techniques in the Methods section.*
☒	☐	A description of all covariates tested
☐	☒	A description of any assumptions or corrections, such as tests of normality and adjustment for multiple comparisons
☐	☒	A full description of the statistical parameters including central tendency (e.g. means) or other basic estimates (e.g. regression coefficient) AND variation (e.g. standard deviation) or associated estimates of uncertainty (e.g. confidence intervals)
☐	☒	For null hypothesis testing, the test statistic (e.g. F, t, r) with confidence intervals, effect sizes, degrees of freedom and P value noted<br>*Give P values as exact values whenever suitable.*
☒	☐	For Bayesian analysis, information on the choice of priors and Markov chain Monte Carlo settings
☒	☐	For hierarchical and complex designs, identification of the appropriate level for tests and full reporting of outcomes
☒	☐	Estimates of effect sizes (e.g. Cohen's d, Pearson's r), indicating how they were calculated

Our web collection on statistics for biologists contains articles on many of the points above.

Software and code

Policy information about availability of computer code

Data collection	No new software code was developed.
Data analysis	Softworx (Applied Precision) was used to reconstruct 3D-SIM. The confocal images were analyzed with Image J (ImageJ bundled with 64-bit Java 1.8.0_172, NIH), maris X64 version 9.5.0 (Bitplane Scientific) and ZEN blue (Zeiss) software. The Western blots were analyzed with Image J (NIH). FACS data was analyzed with Kaluza software (Beckman Coulter). All statistical analysis was performed using GraphPad Prism9 and Microsoft Excel. Proteomic data was processed using Proteome Discover 2.1 and searched against a Uniprot reviewed human database.

For manuscripts utilizing custom algorithms or software that are central to the research but not yet described in published literature, software must be made available to editors and reviewers. We strongly encourage code deposition in a community repository (e.g. GitHub). See the Nature Research guidelines for submitting code & software for further information.

Data

Policy information about availability of data

All manuscripts must include a data availability statement. This statement should provide the following information, where applicable:

- Accession codes, unique identifiers, or web links for publicly available datasets
- A list of figures that have associated raw data
- A description of any restrictions on data availability

The authors declare that the all data supporting the findings of this study are available within the paper and in its supplementary information files.
All figures have associated raw data.
Source data (excel files for main data, supplementary information) are provided with this paper.
Proteomic datasets are provided at Source data files and the mass spectrometry proteomics data have been deposited to the ProteomeXchange Consortium via the PRIDE partner repository with the dataset identifier PXD024620 and 10.6019/PXD024620.

Full microscopy images are provided at Source data files. A list of primers is provided in supplementary table1 of Source data files.

Field-specific reporting

Please select the one below that is the best fit for your research. If you are not sure, read the appropriate sections before making your selection.

- [x] Life sciences
- [ ] Behavioural & social sciences
- [ ] Ecological, evolutionary & environmental sciences

For a reference copy of the document with all sections, see nature.com/documents/nr-reporting-summary-flat.pdf

Life sciences study design

All studies must disclose on these points even when the disclosure is negative.

Sample size	We performed at least three independent biological experiments for the reported studies.
Data exclusions	No data were excluded from the analysis.
Replication	We performed at least three independent biological replicate experiments. For each of these biological replicates, we used additional technical replicates for protein quantification assay and qPCR to ensure the reproducibility of our studies. When representative images were shown, they were based on at least three independent replicate experiments.
Randomization	No randomization as these were animal and cell based studies
Blinding	The tested animals had ear tags. We performed all experiments while being blinded to the animal genotype. After completion of experiments, the genotype was revealed and the data was analyzed according to the genotype. Blinding was used during analysis where the data were quantified/measured blind to the treatments.

Reporting for specific materials, systems and methods

We require information from authors about some types of materials, experimental systems and methods used in many studies. Here, indicate whether each material, system or method listed is relevant to your study. If you are not sure if a list item applies to your research, read the appropriate section before selecting a response.

Materials & experimental systems

n/a	Involved in the study	
	x	Antibodies
	x	Eukaryotic cell lines
x		Palaeontology and archaeology
	x	Animals and other organisms
x		Human research participants
x		Clinical data
x		Dual use research of concern

Methods

n/a	Involved in the study	
x		ChIP-seq
	x	Flow cytometry
x		MRI-based neuroimaging

Antibodies

Antibodies used	Mfn2 (abcam, ab56889, mouse), Mfn2 (Proteintech, 12186-1-AP, rabbit), Mfn1 (Cell signaling, CST-14739, rabbit), Tom20 (Santa Cruz, sc-14415, rabbit), Tom20 (Santa Cruz, sc-17764, mouse), Drp1 (Santa Cruz, sc-271583), Opa1 (Cell signaling, CST-80471), VDAC (Cell signaling, CST-4661, rabbit), COXIV (Proteintech, 11242-1-AP, rabbit), VE-cadherin (Santa Cruz, SC-9989, mouse), VE-cadherin (Cayman #160840, rabbit), b-catenin (abcam; ab32572, Santa Cruz; SC-7963, rabbit), phospho-b-catenin (CST-9561), PECM-1(BD pharminogen 553370, rat), GAPDH (Proteintech, 10494-1-AP, rabbit), P84 (Santa Cruz, sc-514123, mouse), Na+/K+-ATPase(Santa Cruz, sc-21712, mouse), Alexa Fluor647 mouse anti-human CD144 (BD Bioscience, BD561567), anti-Rabbit IgG (H+L) Highly Cross-Adsorbed Secondary Antibody, Alexa Fluor Plus 488(Invitrogen, A32731) or 546(Invitrogen, A11030) or 594 (Invitrogen, A32740), anti-mouse Alexa Fluor 568(Invitrogen, A11031), anti-CD45 (Biolegend, A20), anti-CD11c (Biolegend, N418), anti-Siglec F (Biolegend, S17007L), anti-F4/80 (Biolegend, BM8), anti-CD3 (Biolegend, 17A2),anti-B220 (Biolegend, RA3-6B2), anti-Ly6G (Biolegend, 1A8) and anti-NK1.1 (Biolegend, PK136).
Validation	Antibodies were validated by the commercial vendors and published papers. We additional validated the antibodies ourselves using Western blotting,immunoprecipitation, or immunofluorescence imaging. The detail number of citation according to CiteAb website is follow: Mfn2(ab56889 and 12186-1-AP) was broadly validated (223 and 69 citations). Mfn1 (CST-14739) was broadly validated (27 citations).Tom20 (sc-14415 and sc-17764) was broadly validated (83 and 271 citations). Drp1 (sc-271583) was broadly validated (41 citations). Opa1 (CST-80471) was broadly validated (34 citations).VDAC (CST-4661) was broadly validated (153 citations).COXIV (11242-1-AP) was broadly validated (158 citations). VE-cadherin (SC-9989 and Cayman 160840) was broadly validated (208 and 15 citations). b-catenin (ab32572 and SC-7963) was broadly validated (617 and 643 citations). phospho-b-catenin (CST-9561) was broadly validated (290 citation), PECM-1(BD pharminogen 553370) was broadly validated (540 citations). GAPDH (10494-1-AP) was broadly validated (2413 citations). Na+/K+-ATPase(sc-21712) was broadly validated (128 citations).anti-CD45 (A20), anti-CD11c

(N418), anti-Siglec F (S17007L), anti-F4/80 (BM8), anti-CD3 (17A2),anti-B220 (RA3-6B2), anti-Ly6G (1A8) and anti-NK1.1 (PK136) were broadly validated with 114, 186, 3, 171, 128, 157, 207, 84 citations, respectively.

Eukaryotic cell lines

Policy information about cell lines

Cell line source(s)	We purchased human lung micro-vascular endothelial cells (#CC-2527) from Lonza. We purchased HEK293T (CRL-11268) from ATCC.
Authentication	We used primary endothelial cells that are isolated by the vendor and authenticated by the vendor.<br>We additionally performed VE-cadherin Western blotting to validate their endothelial phenotype.
Mycoplasma contamination	The vendors test negative for mycoplasma, bacteria, yeast, and fungi.
Commonly misidentified lines (See ICLAC register)	We did not use any commonly misidentified cell lines for our experimental studies.

Animals and other organisms

Policy information about studies involving animals; ARRIVE guidelines recommended for reporting animal research

Laboratory animals	C57/BL6, Mfn2fl/fl, Mfn2fl/lf:Cdh5-ERT2-Cre, 8-10weeks age, female and male.<br>Housing condition: Mice were maintained under standard conditions (standard diet and water) at 23 °C and ~60% humidity with 12 h light and 12 h dark cycles.
Wild animals	The study did not involve wild animals.
Field-collected samples	The study did not involve samples collected from the field.
Ethics oversight	All animal studies were carried out following protocols approved by the Animal Care and Institutional Biosafety Committee of the University of Illinois at Chicago. The Biological Resources Laboratory is fully accredited by the American Association for Accreditation of Laboratory Animal Care (AAALAC). All aspects of working with mice, including procurement, quarantine, housing, management, veterinary care and disposal of carcasses follow the guidelines set down in the NIH Guide for the Care and Use of Laboratory Animals.

Note that full information on the approval of the study protocol must also be provided in the manuscript.

Flow Cytometry

Plots

Confirm that:

- [x] The axis labels state the marker and fluorochrome used (e.g. CD4-FITC).
- [x] The axis scales are clearly visible. Include numbers along axes only for bottom left plot of group (a 'group' is an analysis of identical markers).
- [x] All plots are contour plots with outliers or pseudocolor plots.
- [x] A numerical value for number of cells or percentage (with statistics) is provided.

Methodology

Sample preparation	Single cell suspensions were isolated from the whole lung tissue of Mfn2fl/fl and Mfn2EC-/- mice.
Instrument	Gallios flow cytometer (Beckman Coulter, Pasadena, CA)
Software	Kaluza software (Beckman Coulter)
Cell population abundance	We determined the relevant cell population based on cell surface markers after gating on the CD45 positive leukocyte population. Each cell population is presented as % of whole lung cells; Neutrophil (4~20%), DC (1~2%), Interstitial macrophage2 (2~6%), monocyte (0.5~1%), interstitial macrophage1 (0.1~0.2%), B cell (3~14%), B+T cell (0.3~95), NKT (3~18%), and T cell (8~23%).
Gating strategy	Single cells isolated from lung tissue were gated on CD45 positivity and then analyzed by FACS for the following cell identities: Alveolar macrophage (CD11c+SiglecF+), eosiohphil (CD11c-SiglecF+), neutrophil (CD11c-siglecF-Ly6G+), interstitial macrophage1 (CD11c-siglecF-F4/80+), monocyte (CD11c-SiglecF-F4/80-,Ly6G-), Interstitial macrophage2 (CD11c+SiglecF-F4/80+), DC (CD11c+siglecF-F4/80-), T cells (CD3+B220-), B cells (CD3-B220+), B+T cells (CD3+B220+) and NK T (CD3+B220-NK1.1+).

- [x] Tick this box to confirm that a figure exemplifying the gating strategy is provided in the Supplementary Information.
