# Supplementary material for: Mitofusin-2 stabilizes adherens junctions and suppresses endothelial inflammation via modulation of β-catenin signaling
Source: Nat Commun. 2021 May 12;12:2736. doi: 10.1038/s41467-021-23047-6 (PMC8115264; doi:10.1038/s41467-021-23047-6)
Supplement: Supplementary file 4 — Source Data [file 41467_2021_23047_MOESM4_ESM.zip › Source Data files/Full microscopy image data set for Main Data (NCOMMS-19-32607C).pptx]

## Slide 1
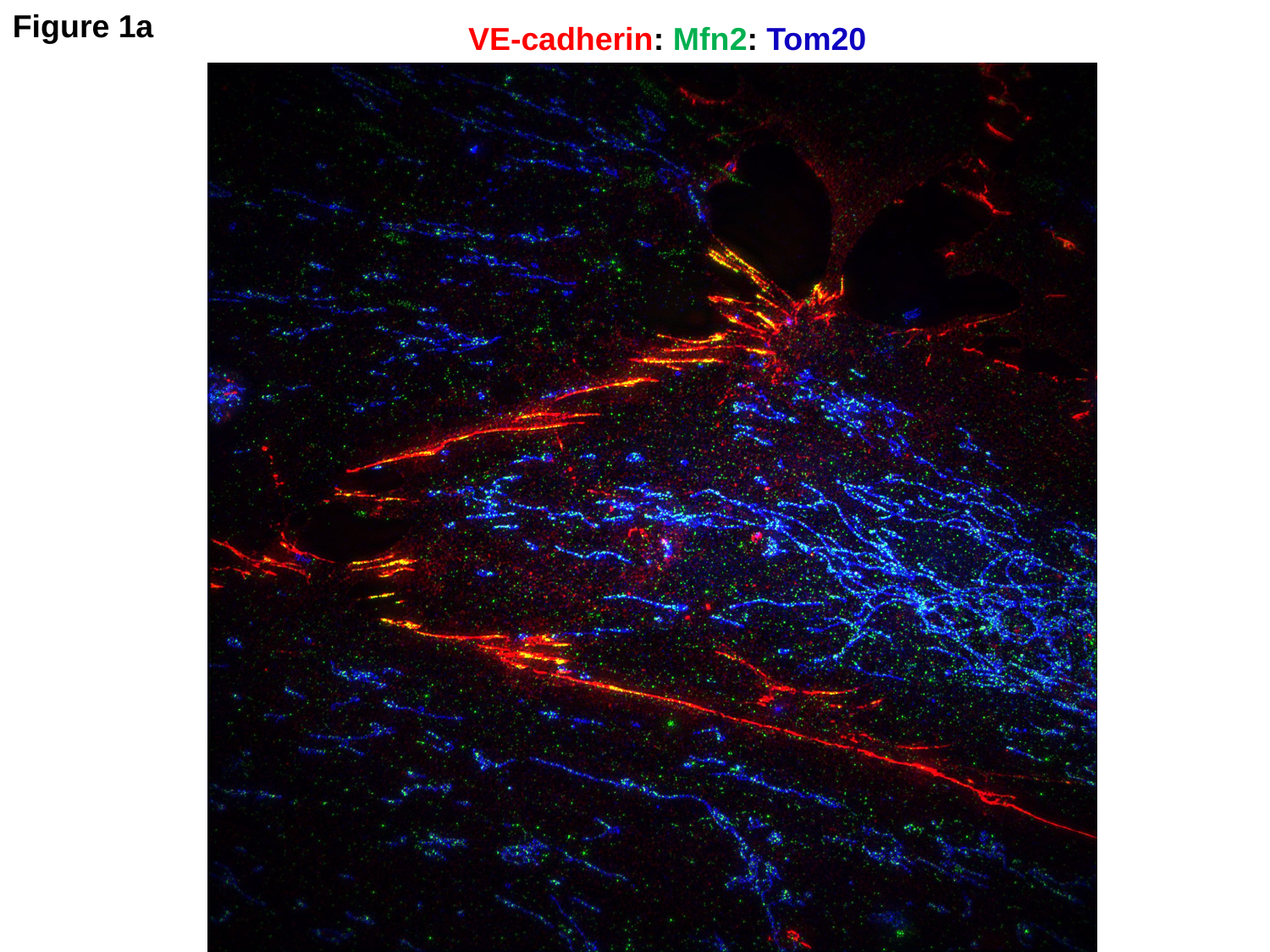

Figure 1a
VE-cadherin: Mfn2: Tom20

## Slide 2
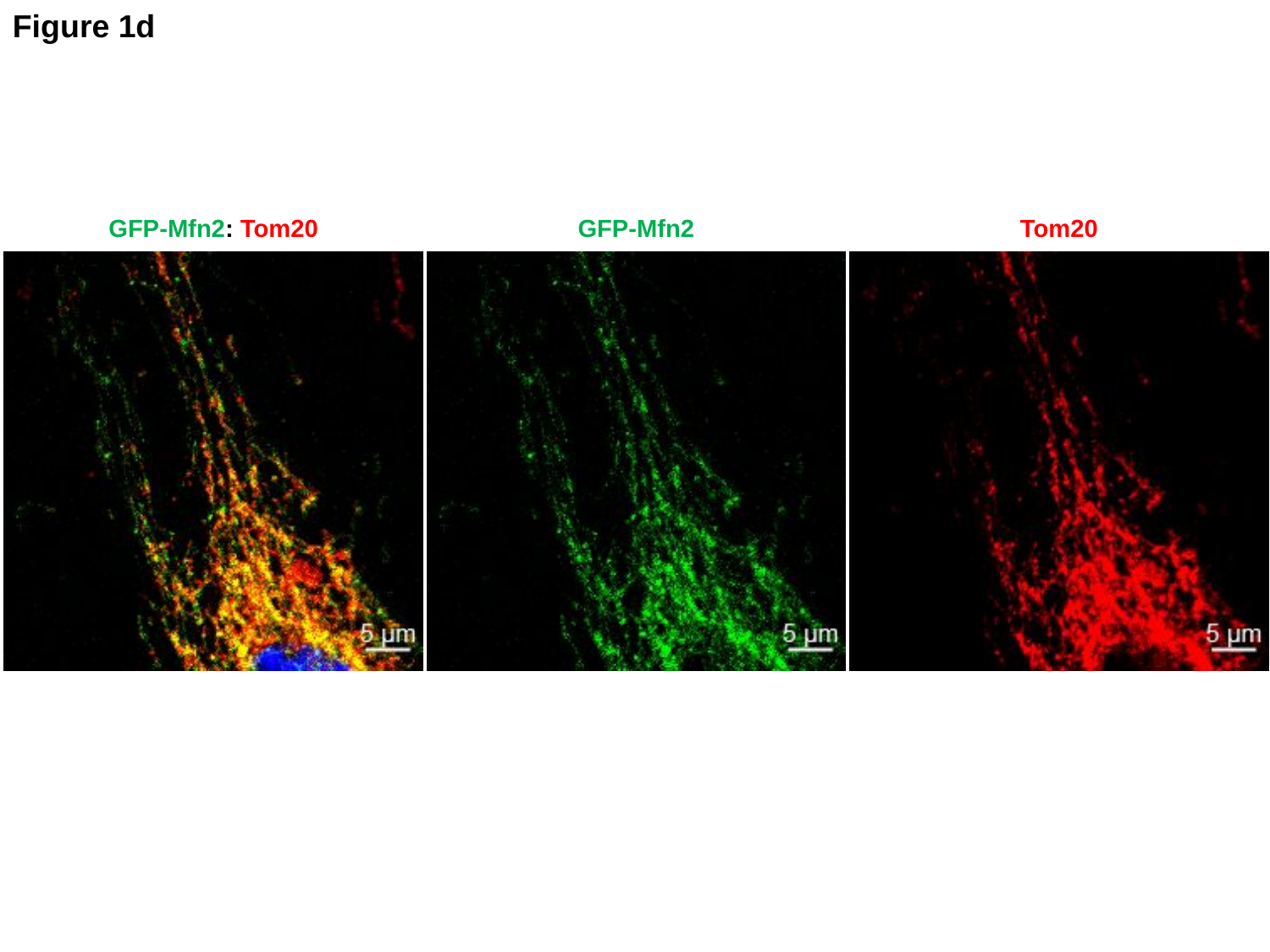

Figure 1d
GFP-Mfn2: Tom20
GFP-Mfn2
Tom20

## Slide 3
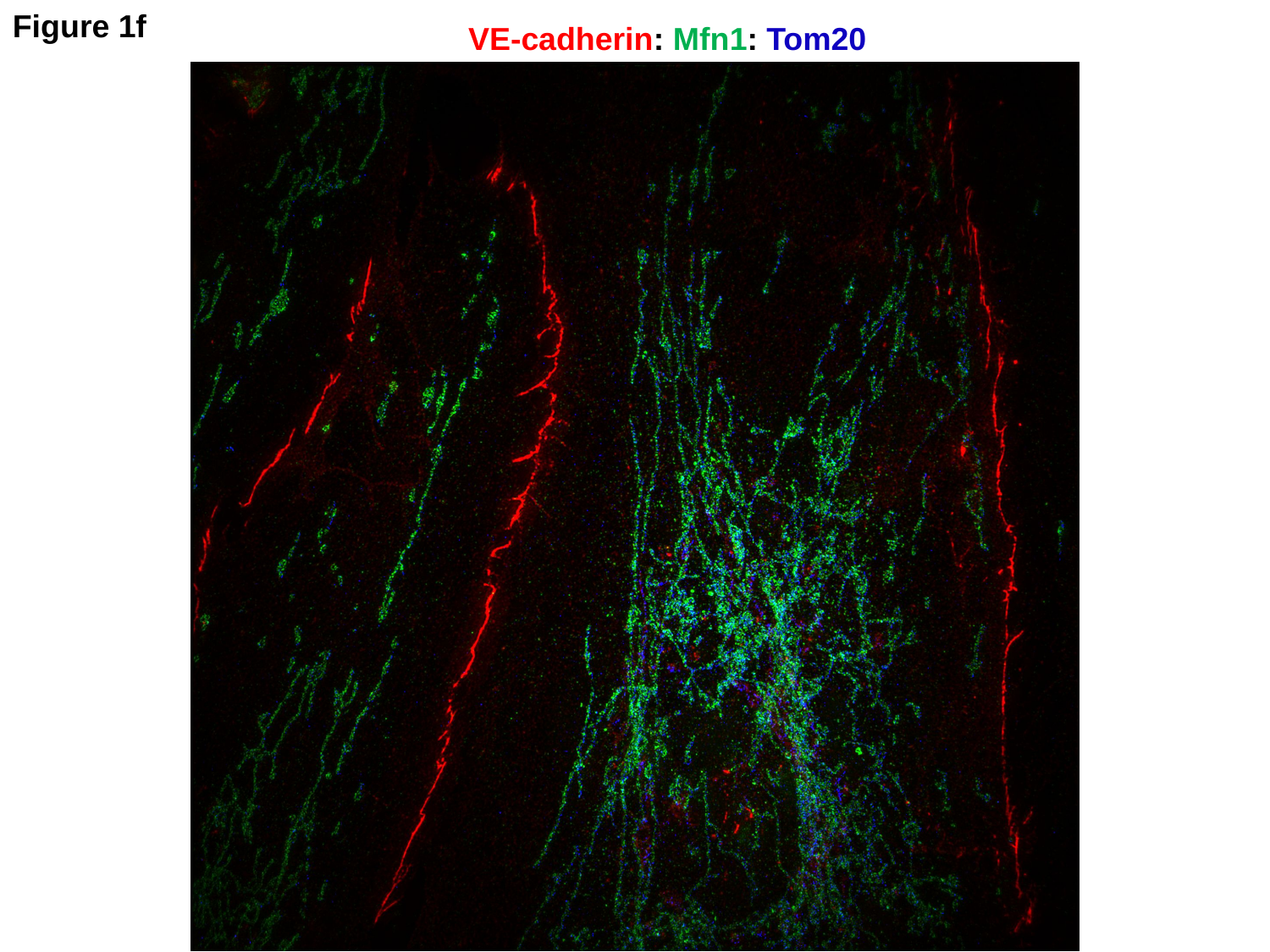

Figure 1f
VE-cadherin: Mfn1: Tom20

## Slide 4
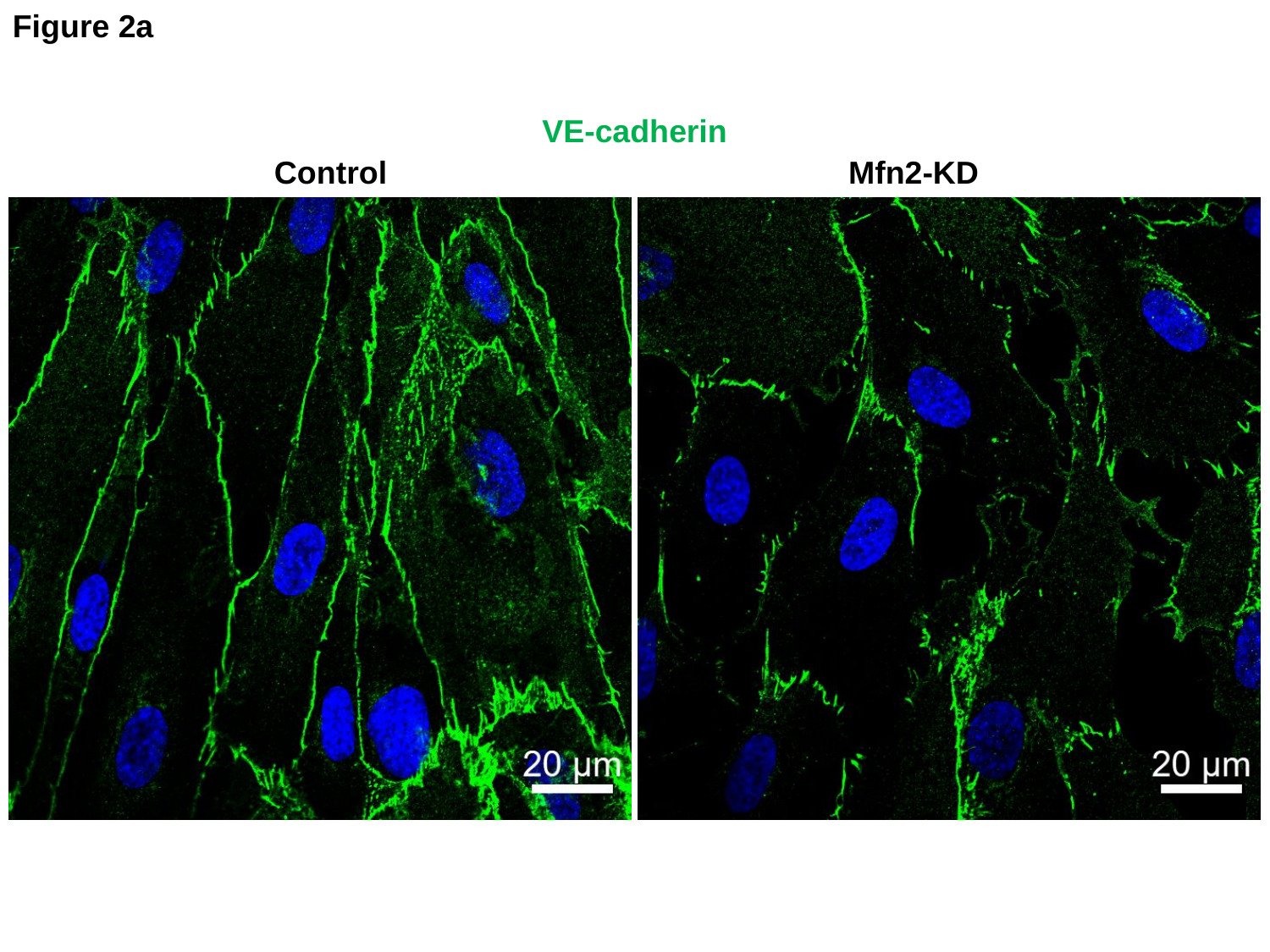

Figure 2a
VE-cadherin
Control
Mfn2-KD

## Slide 5
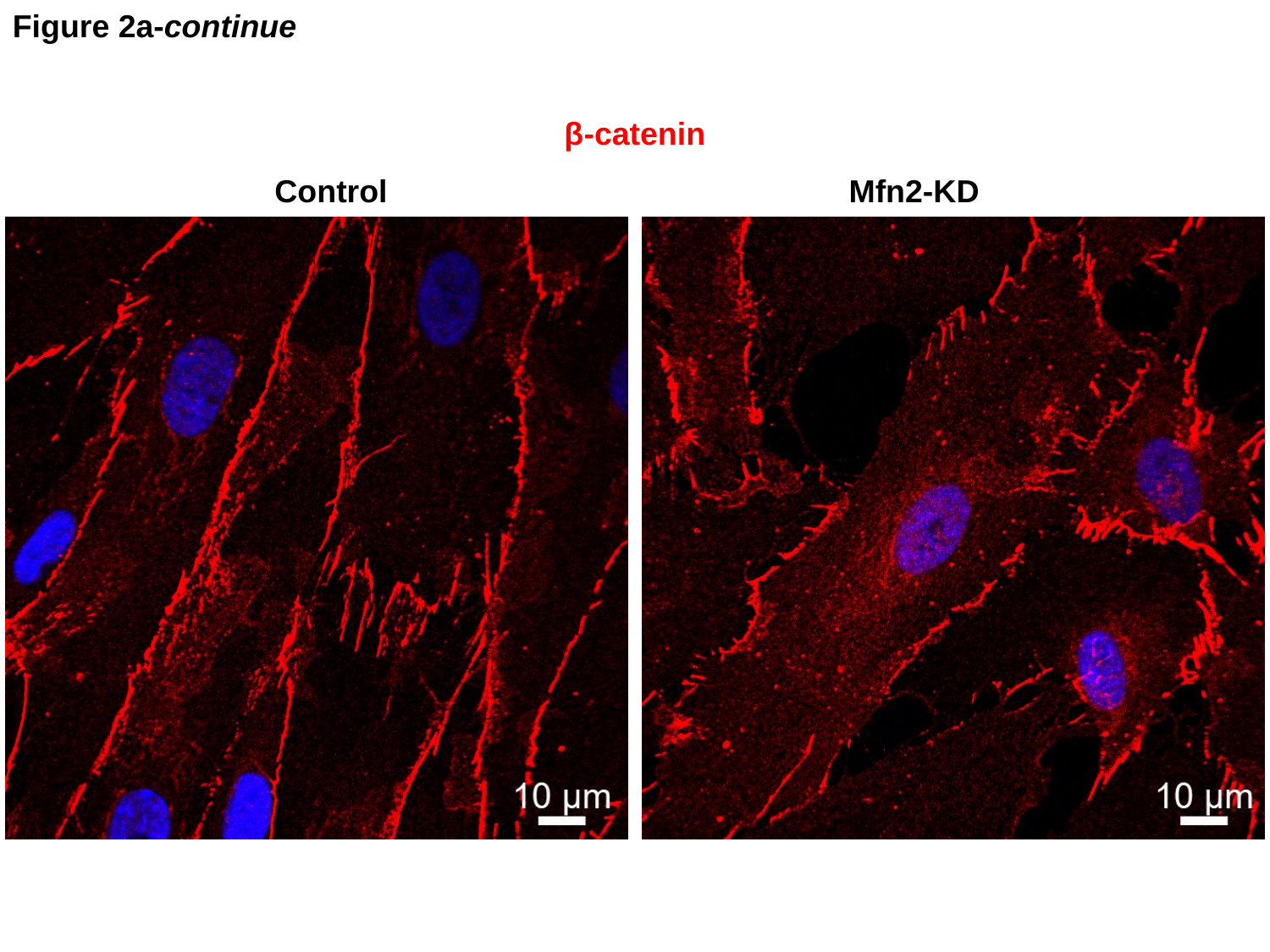

Figure 2a-continue
β-catenin
Control
Mfn2-KD

## Slide 6
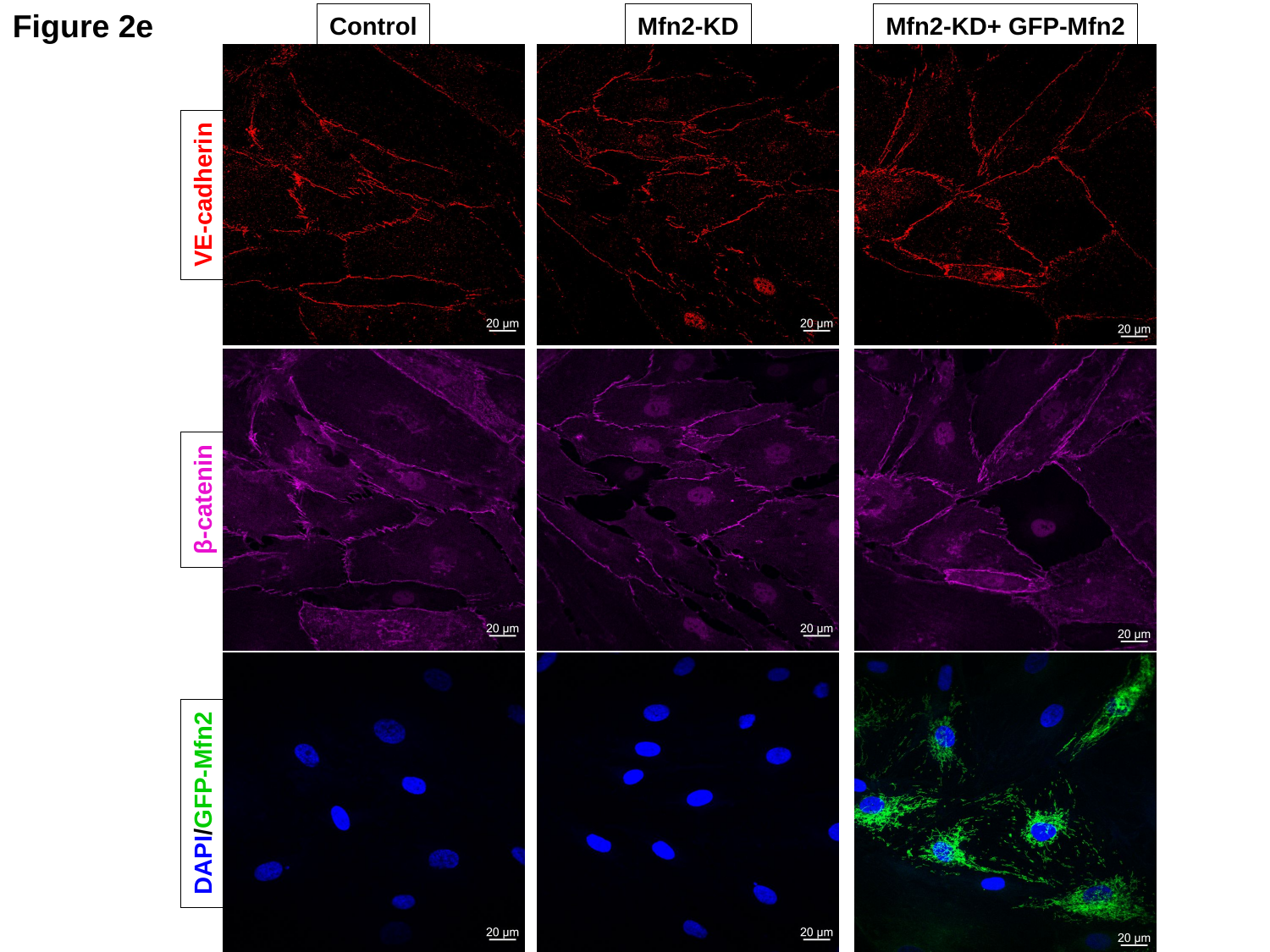

Figure 2e
Control
Mfn2-KD
Mfn2-KD+ GFP-Mfn2
VE-cadherin
β-catenin
DAPI/GFP-Mfn2

## Slide 7
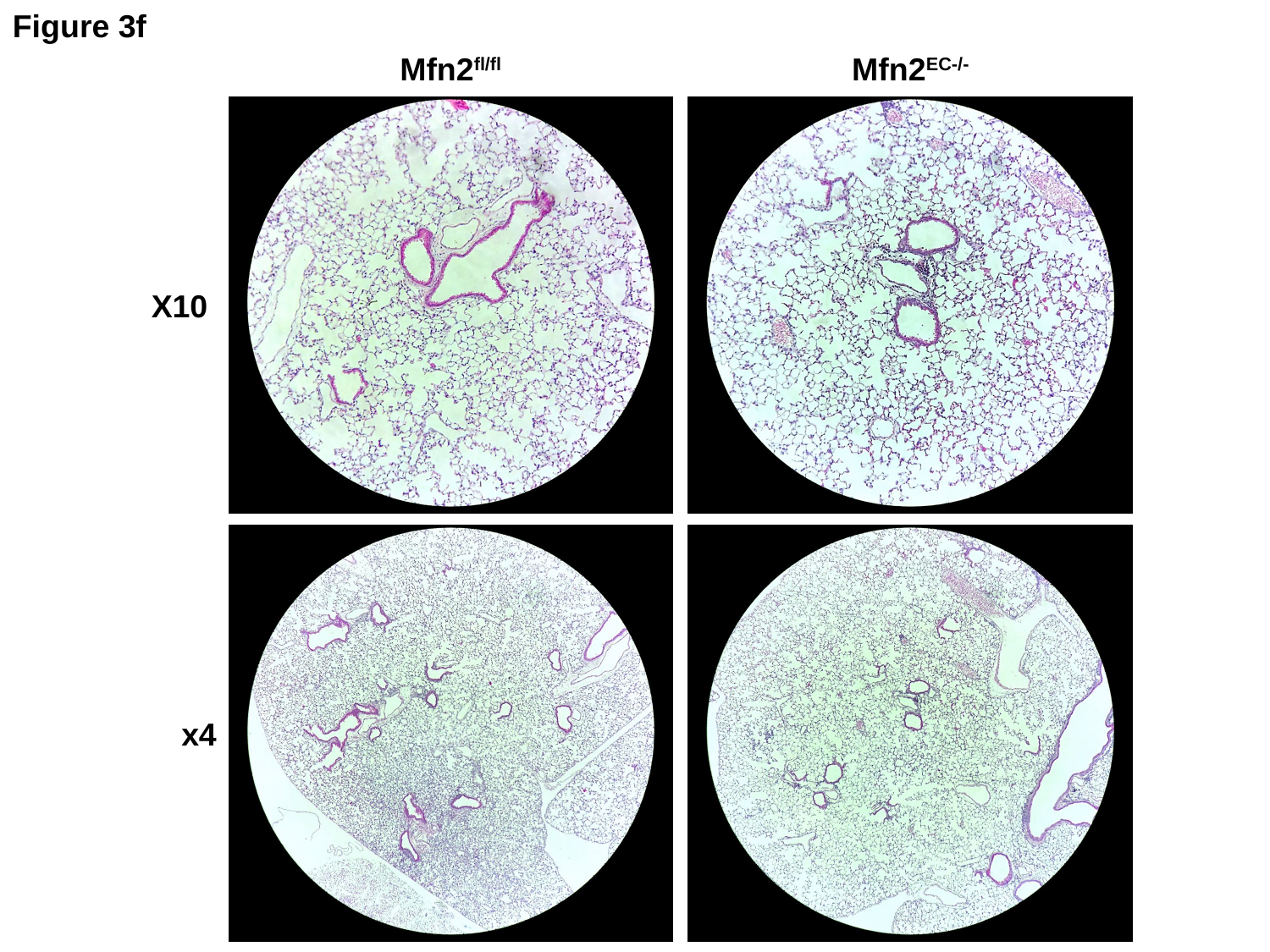

Figure 3f
Mfn2fl/fl
Mfn2EC-/-
X10
x4

## Slide 8
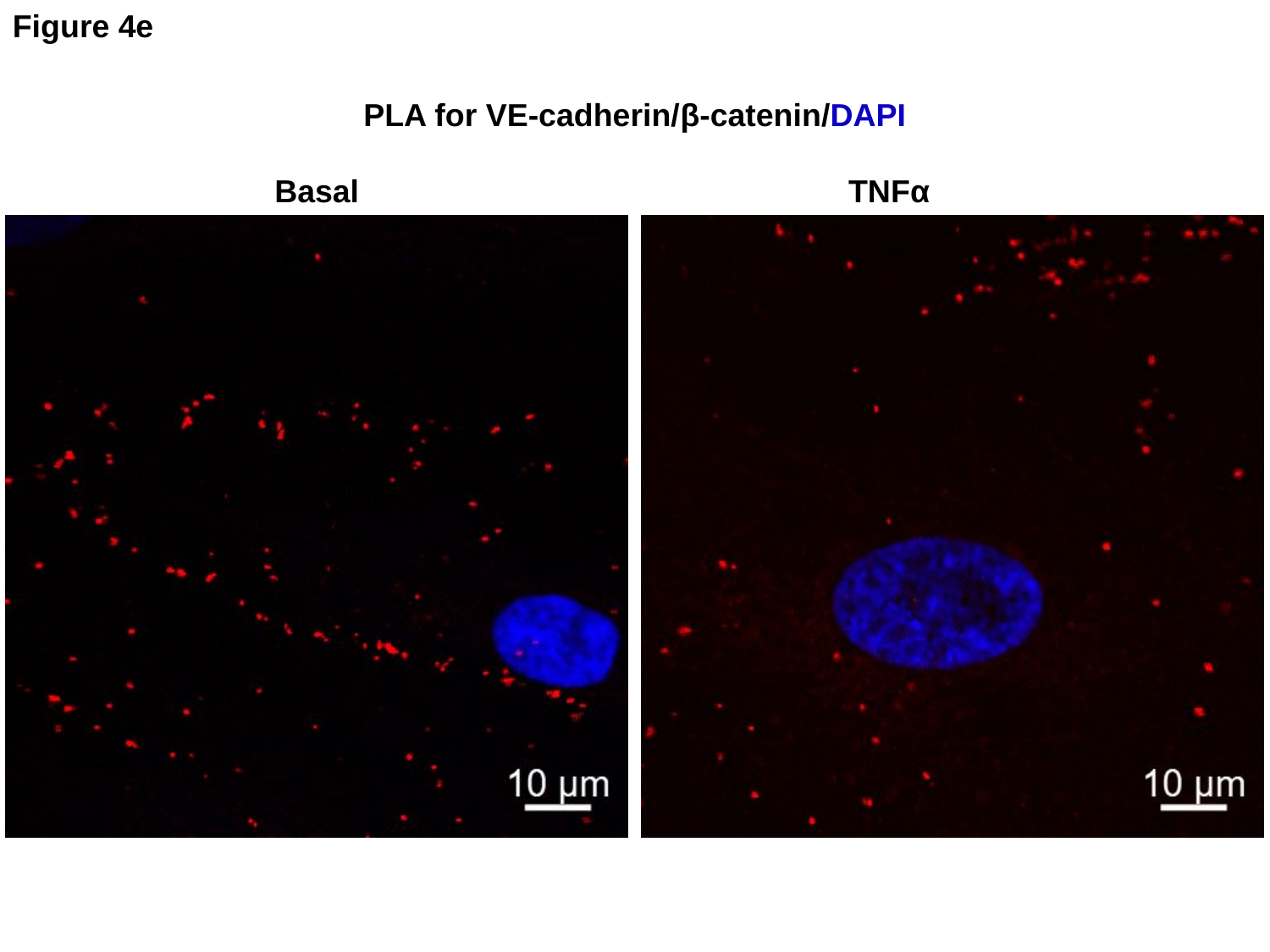

Figure 4e
PLA for VE-cadherin/β-catenin/DAPI
Basal
TNFα

## Slide 9
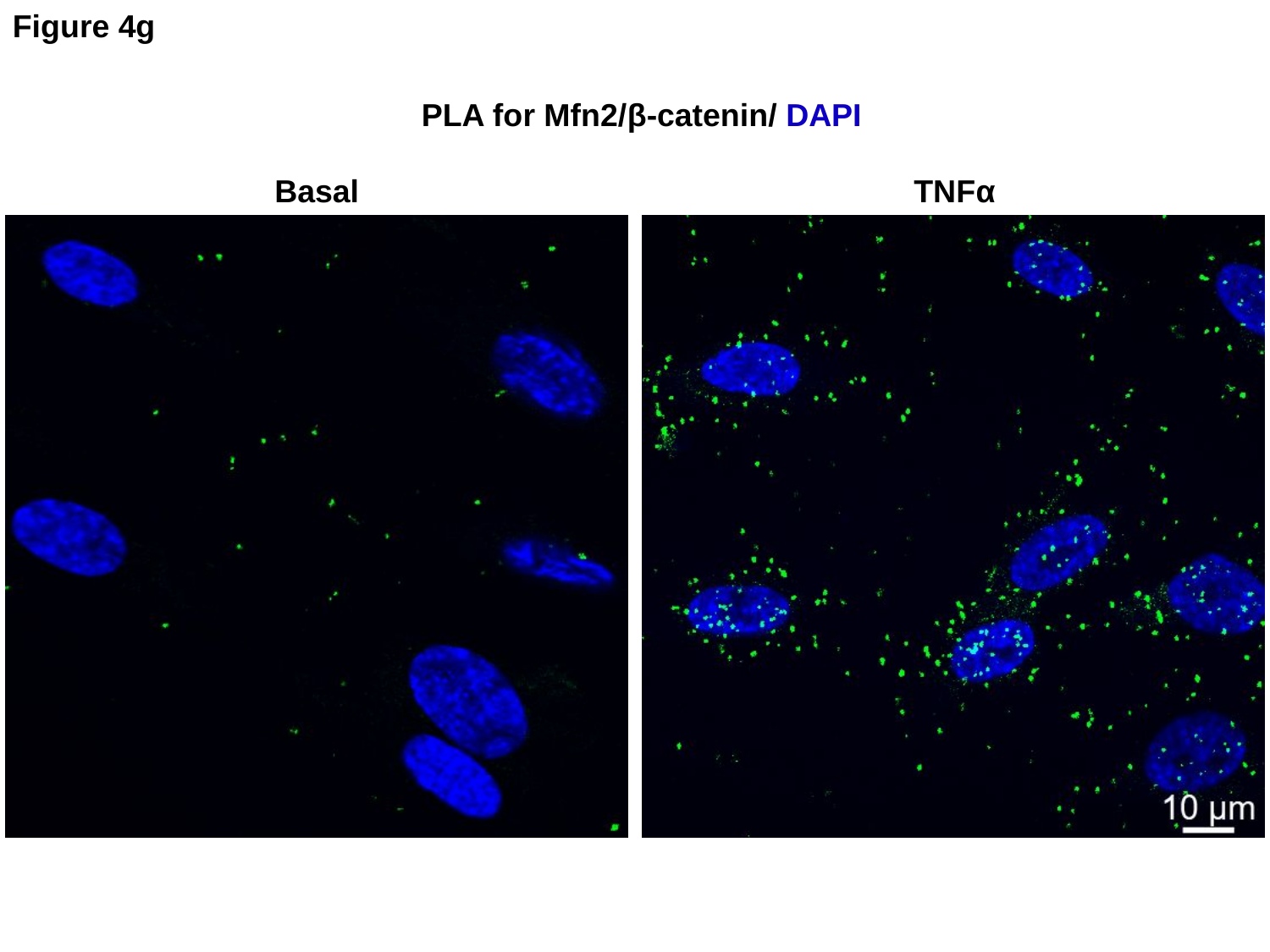

Figure 4g
PLA for Mfn2/β-catenin/ DAPI
Basal
TNFα

## Slide 10
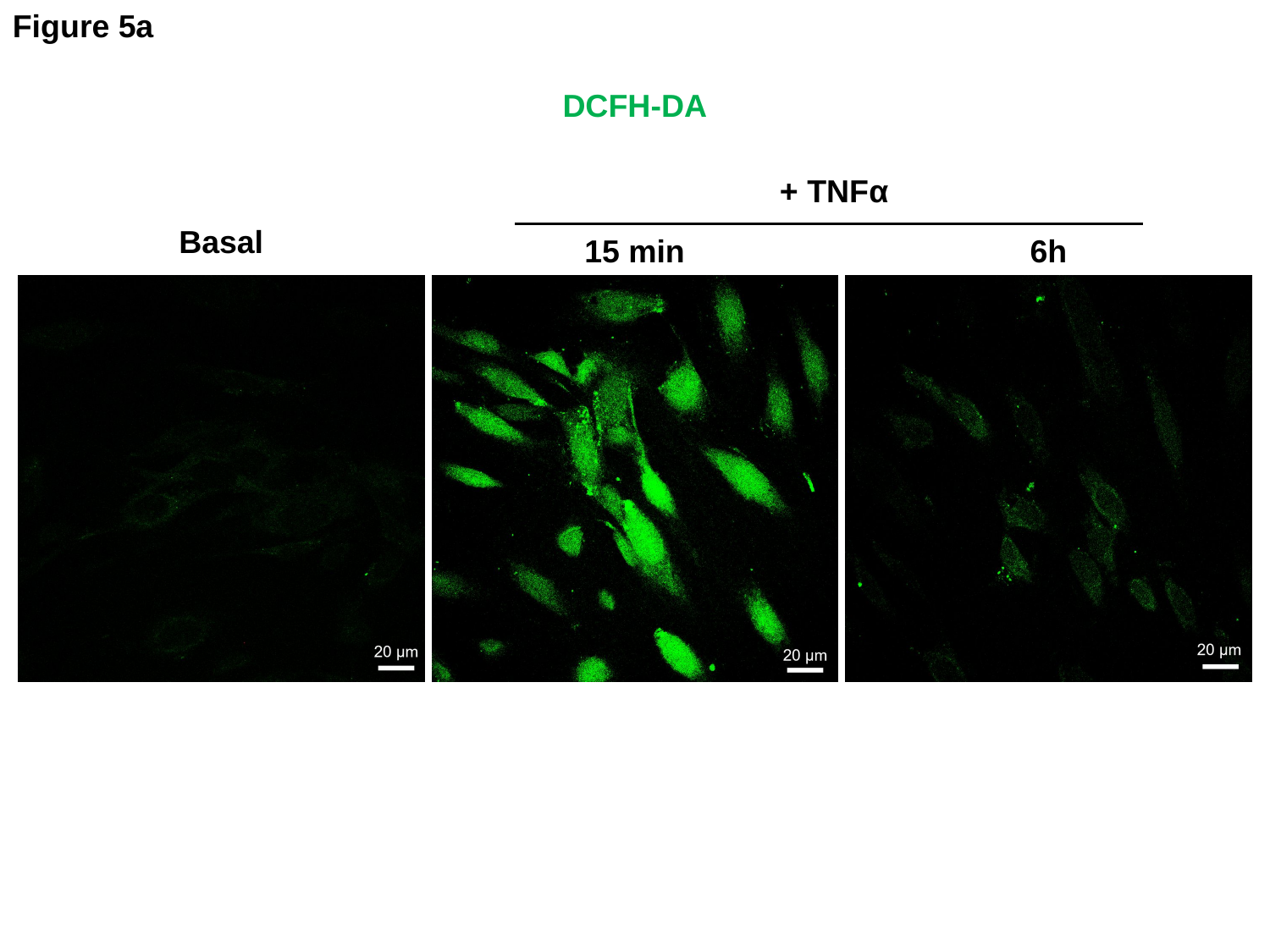

Figure 5a
DCFH-DA
+ TNFα
Basal
15 min
6h

## Slide 11
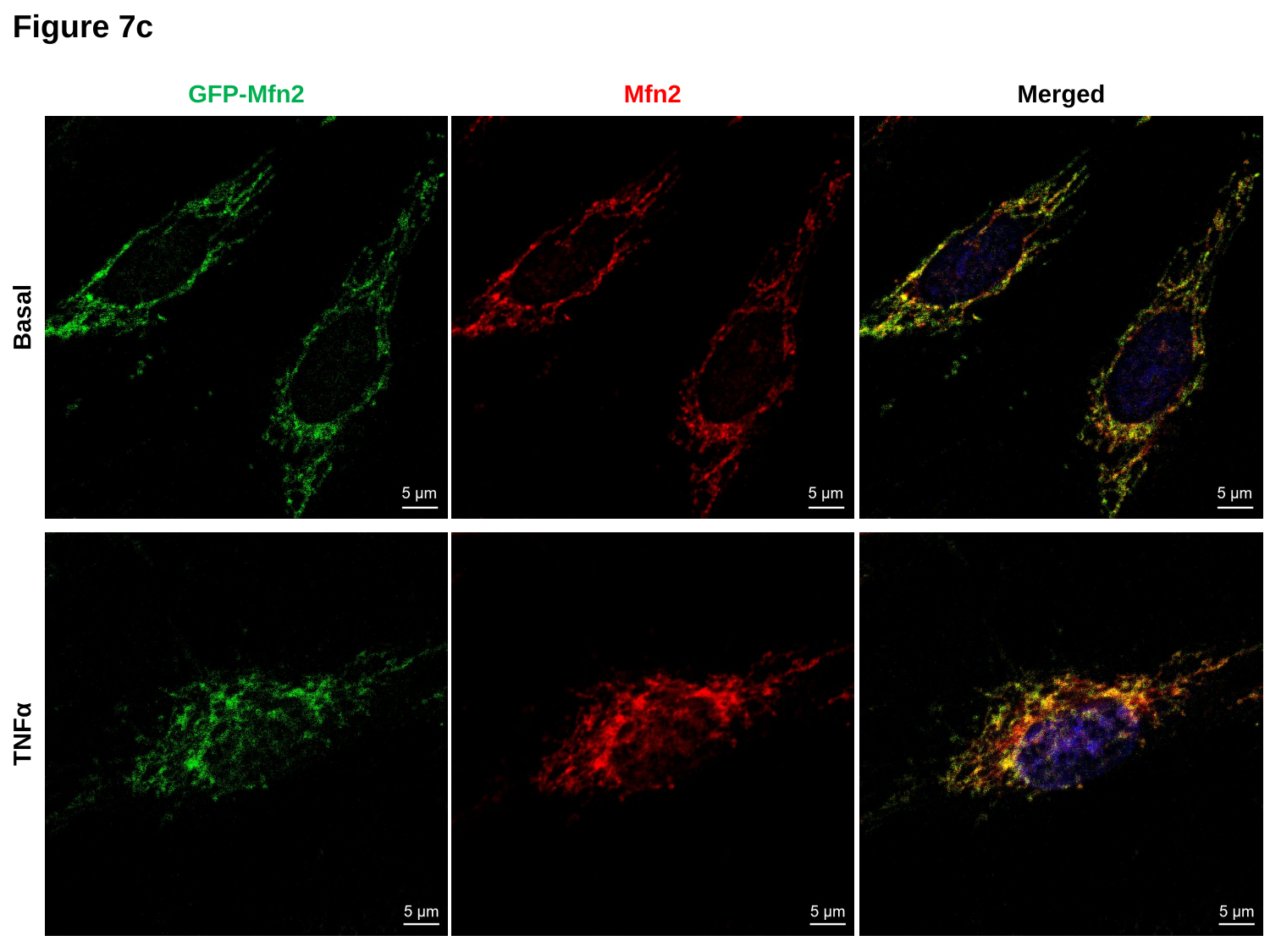

Figure 7c
GFP-Mfn2
Mfn2
Merged
Basal
TNFα

## Slide 12
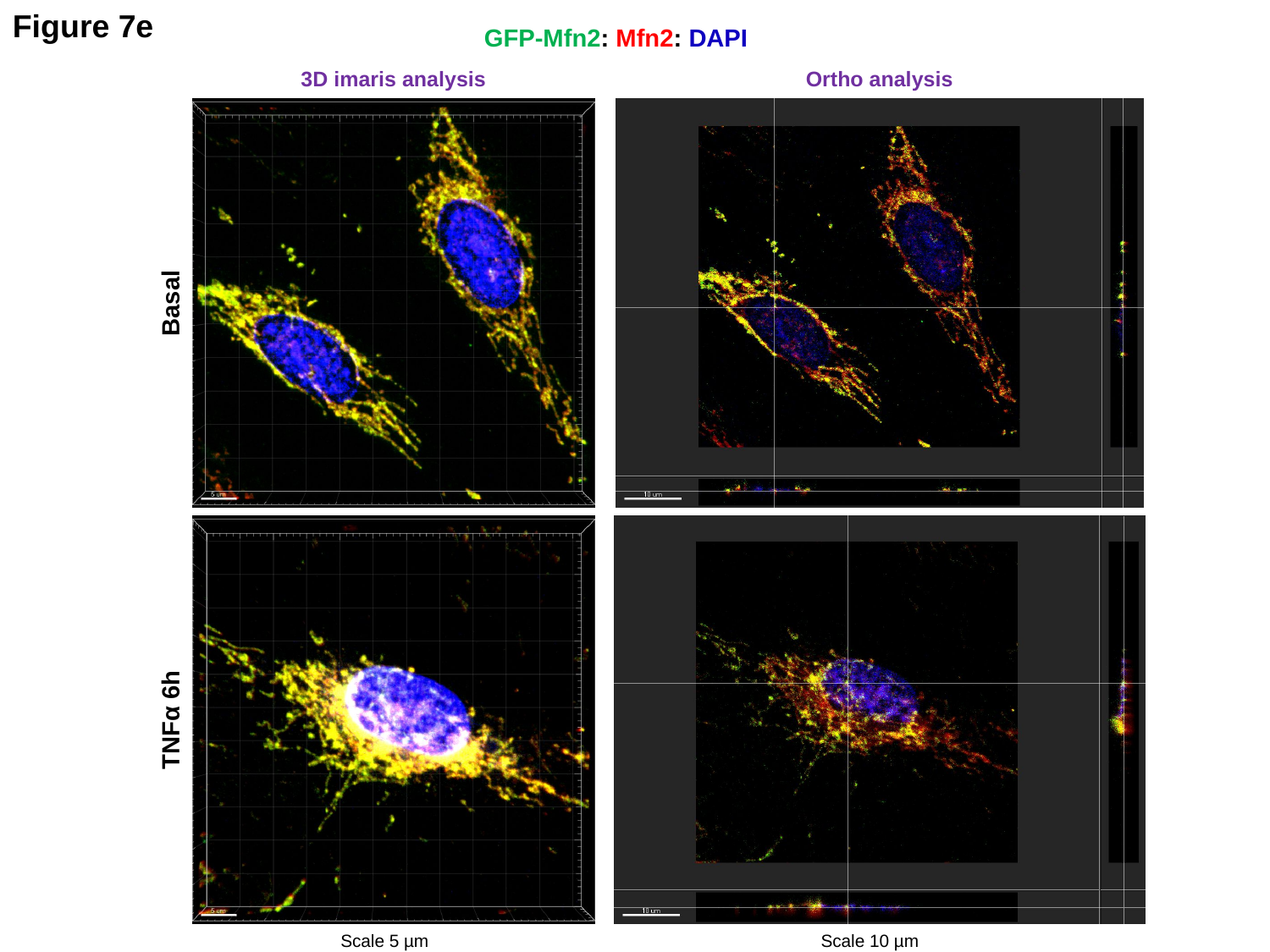

Figure 7e
GFP-Mfn2: Mfn2: DAPI
3D imaris analysis
Ortho analysis
Basal
TNFα 6h
Scale 5 µm
Scale 10 µm

## Slide 13
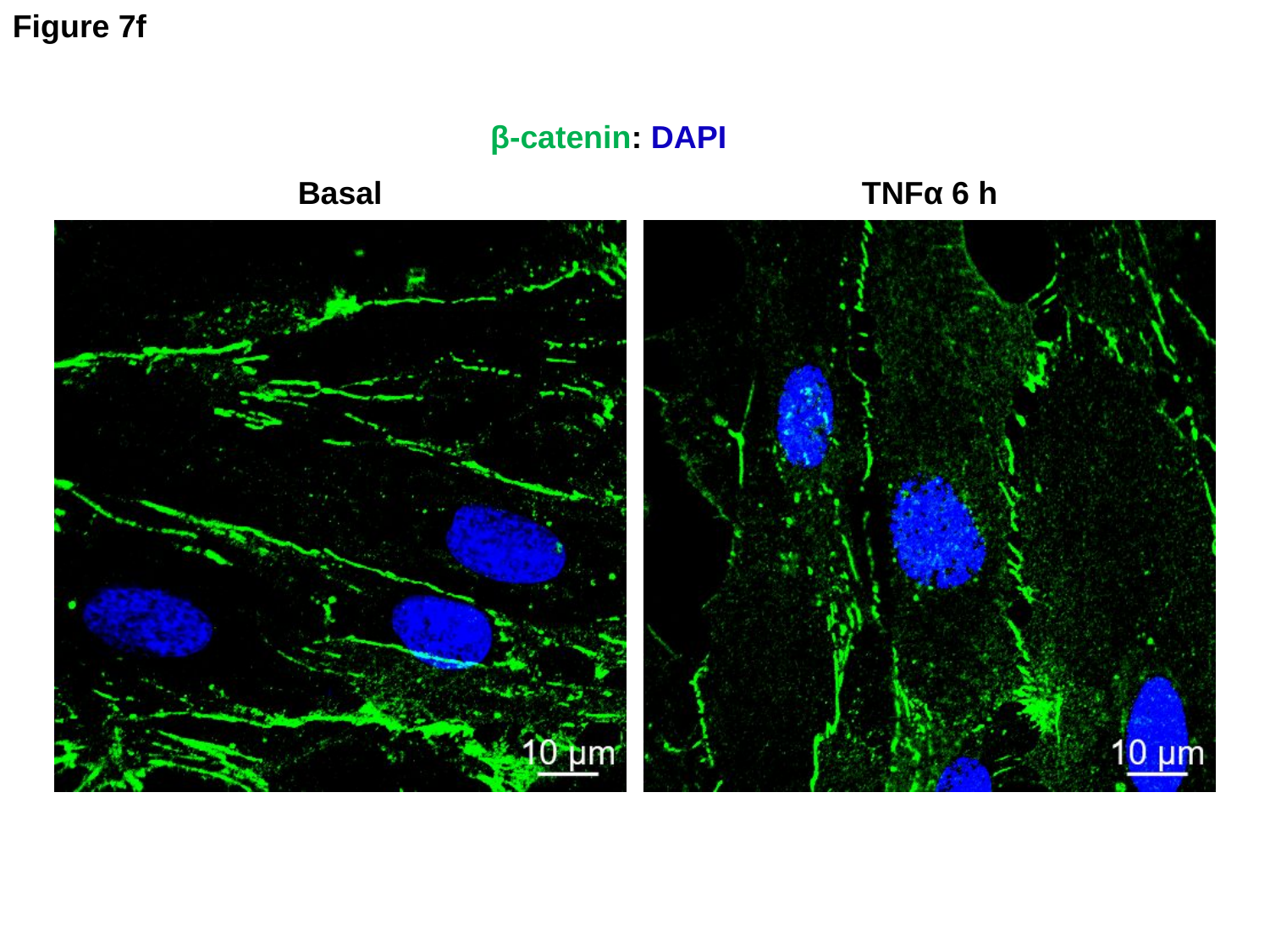

Figure 7f
β-catenin: DAPI
Basal
TNFα 6 h
