# Supplementary material for: Mitofusin-2 stabilizes adherens junctions and suppresses endothelial inflammation via modulation of β-catenin signaling
Source: Nat Commun. 2021 May 12;12:2736. doi: 10.1038/s41467-021-23047-6 (PMC8115264; doi:10.1038/s41467-021-23047-6)
Supplement: Supplementary file 4 — Source Data [file 41467_2021_23047_MOESM4_ESM.zip › Source Data files/Full microscopy image data set for supp Info (NCOMMS-19-32607C).pptx]

## Slide 1
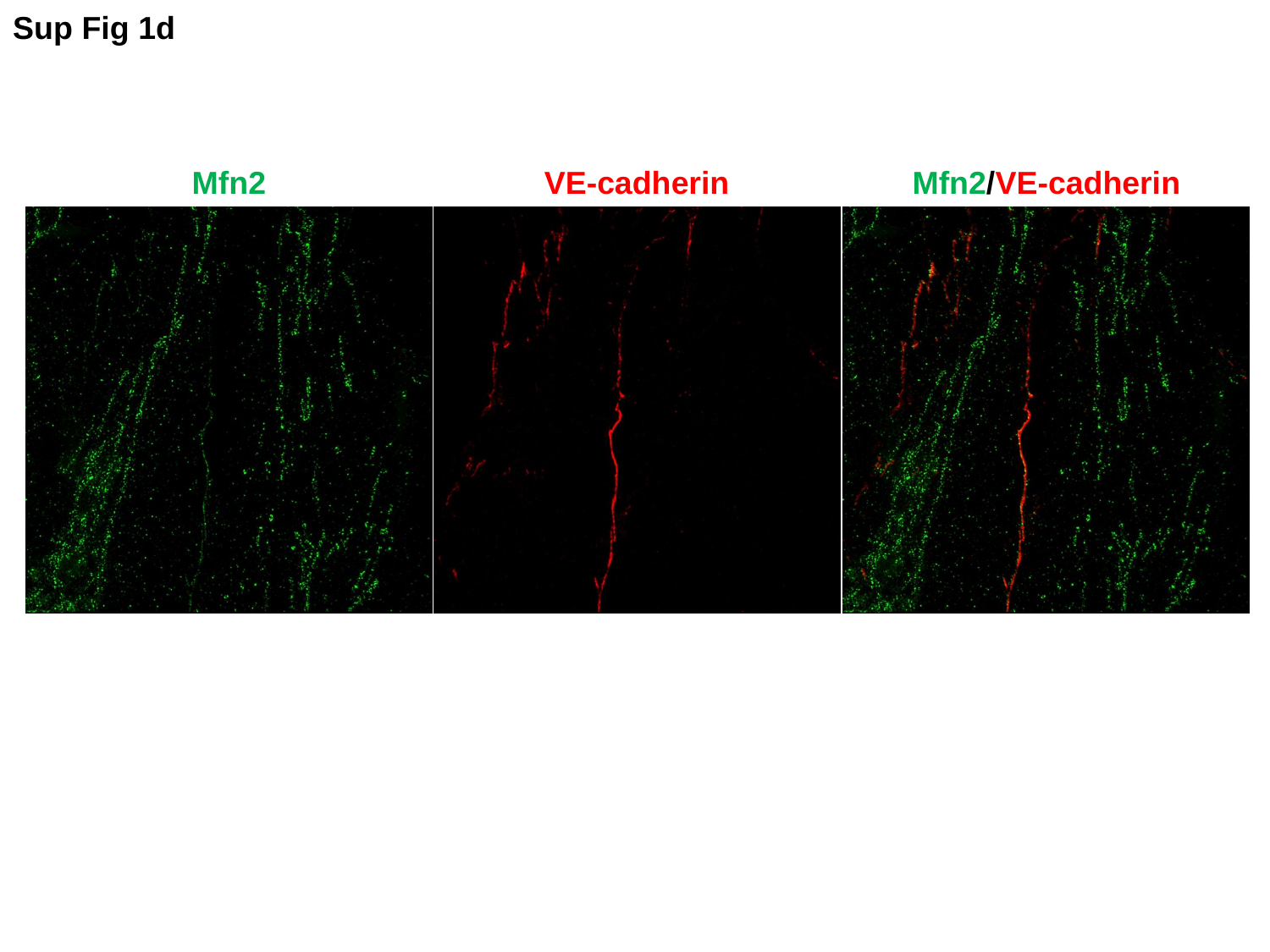

Sup Fig 1d
Mfn2
VE-cadherin
Mfn2/VE-cadherin

## Slide 2
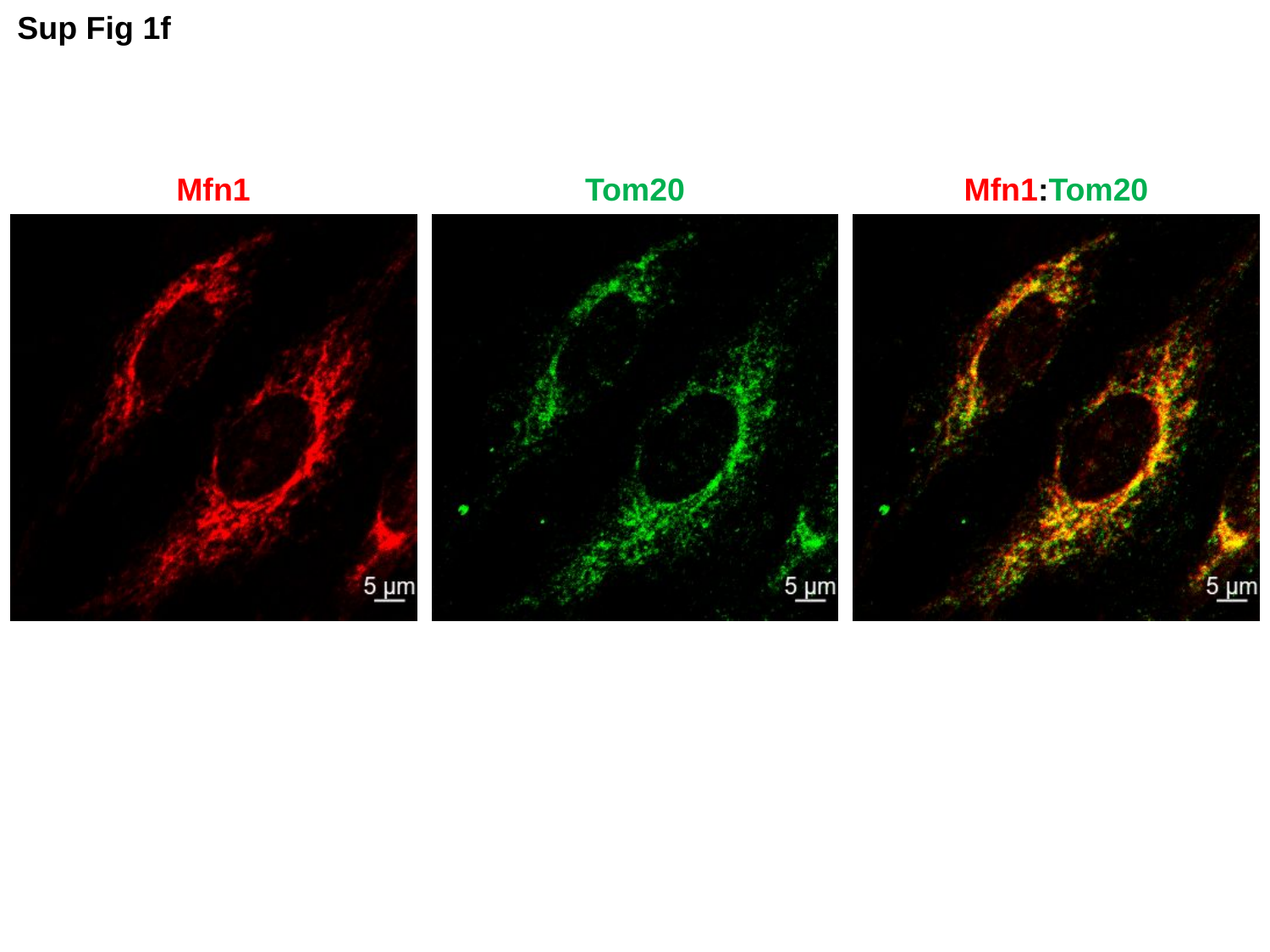

Sup Fig 1f
Mfn1
Tom20
Mfn1:Tom20

## Slide 3
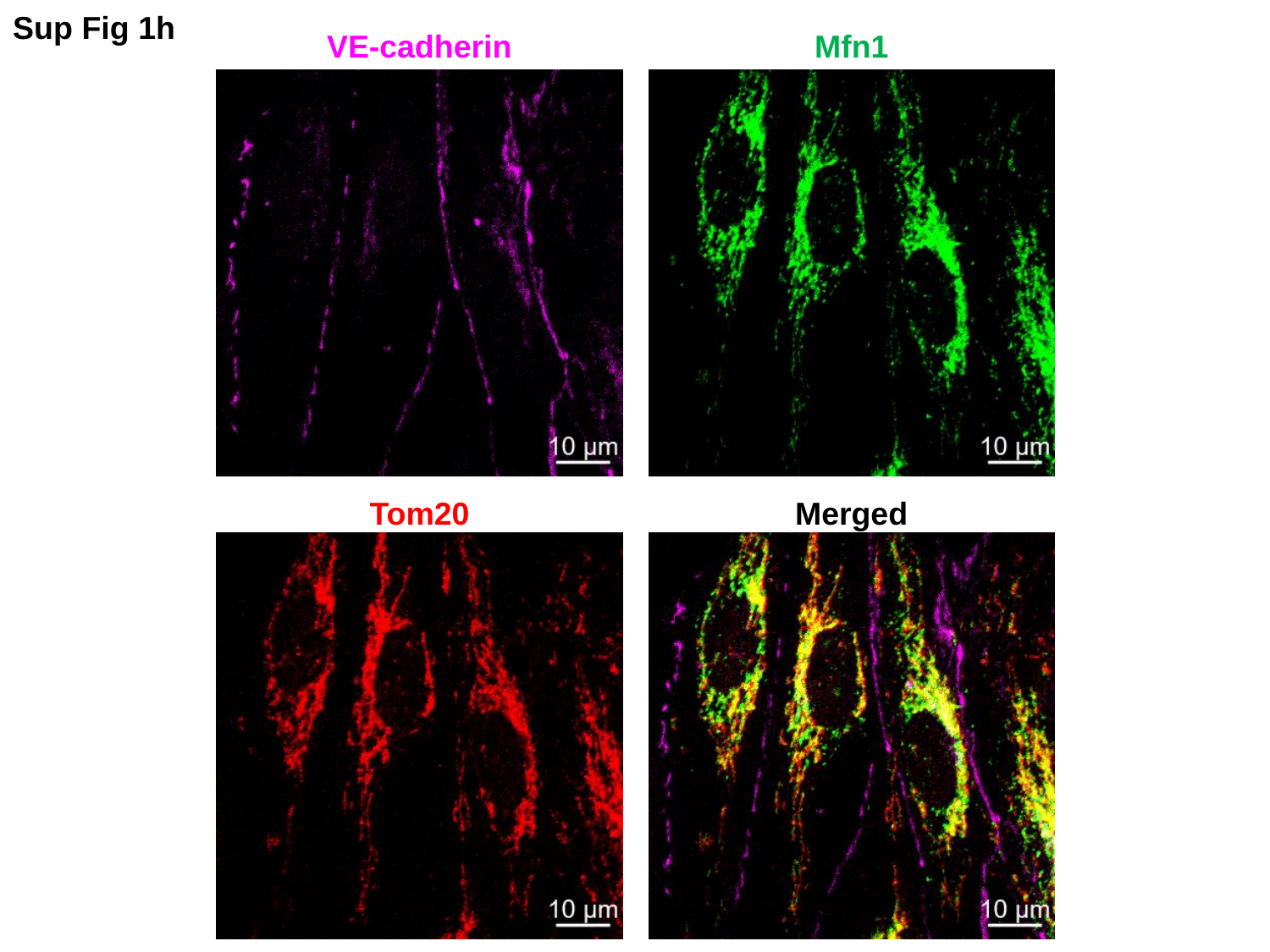

Sup Fig 1h
VE-cadherin
Mfn1
Tom20
Merged

## Slide 4
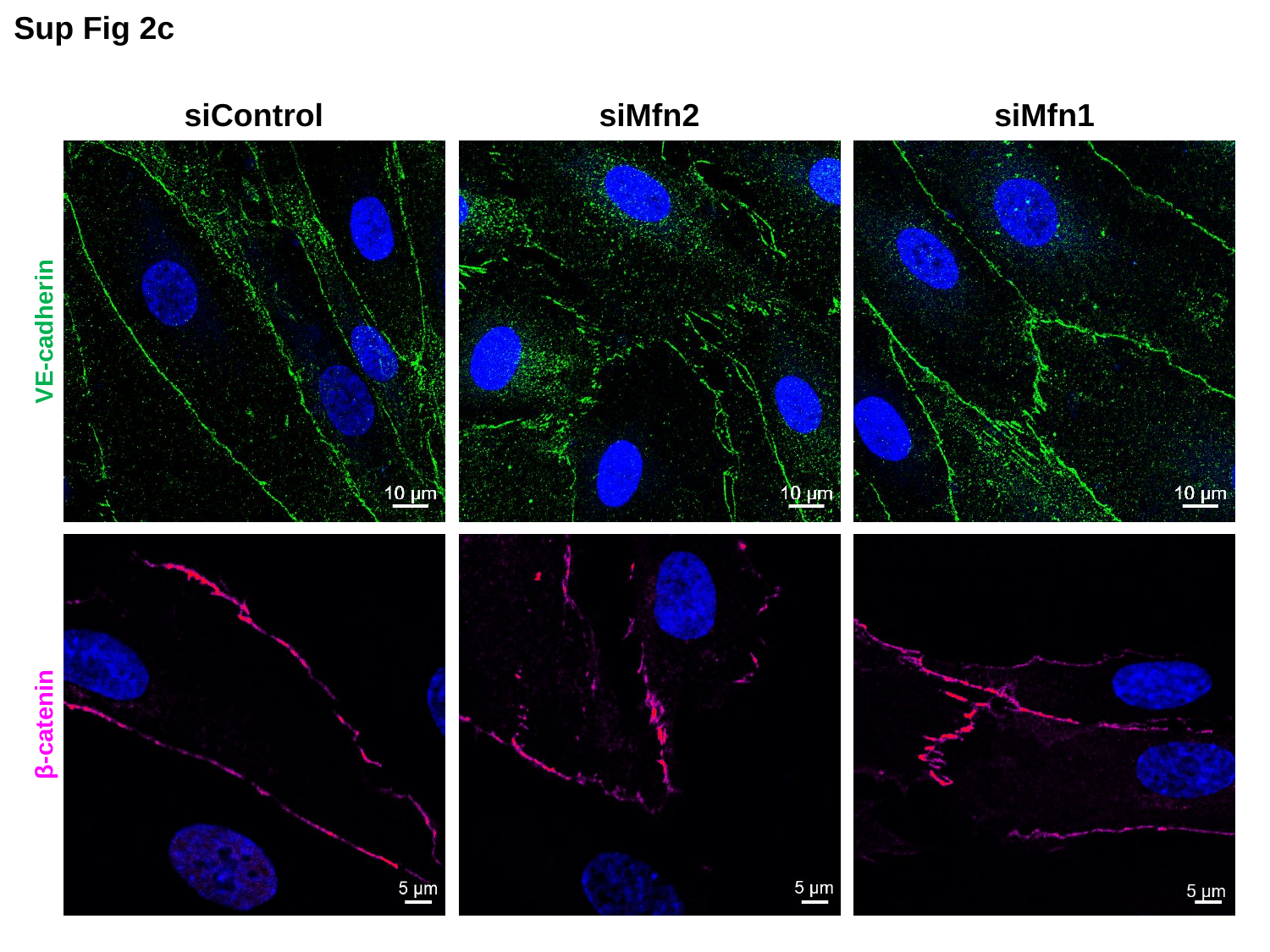

Sup Fig 2c
siControl
siMfn2
siMfn1
VE-cadherin
β-catenin

## Slide 5
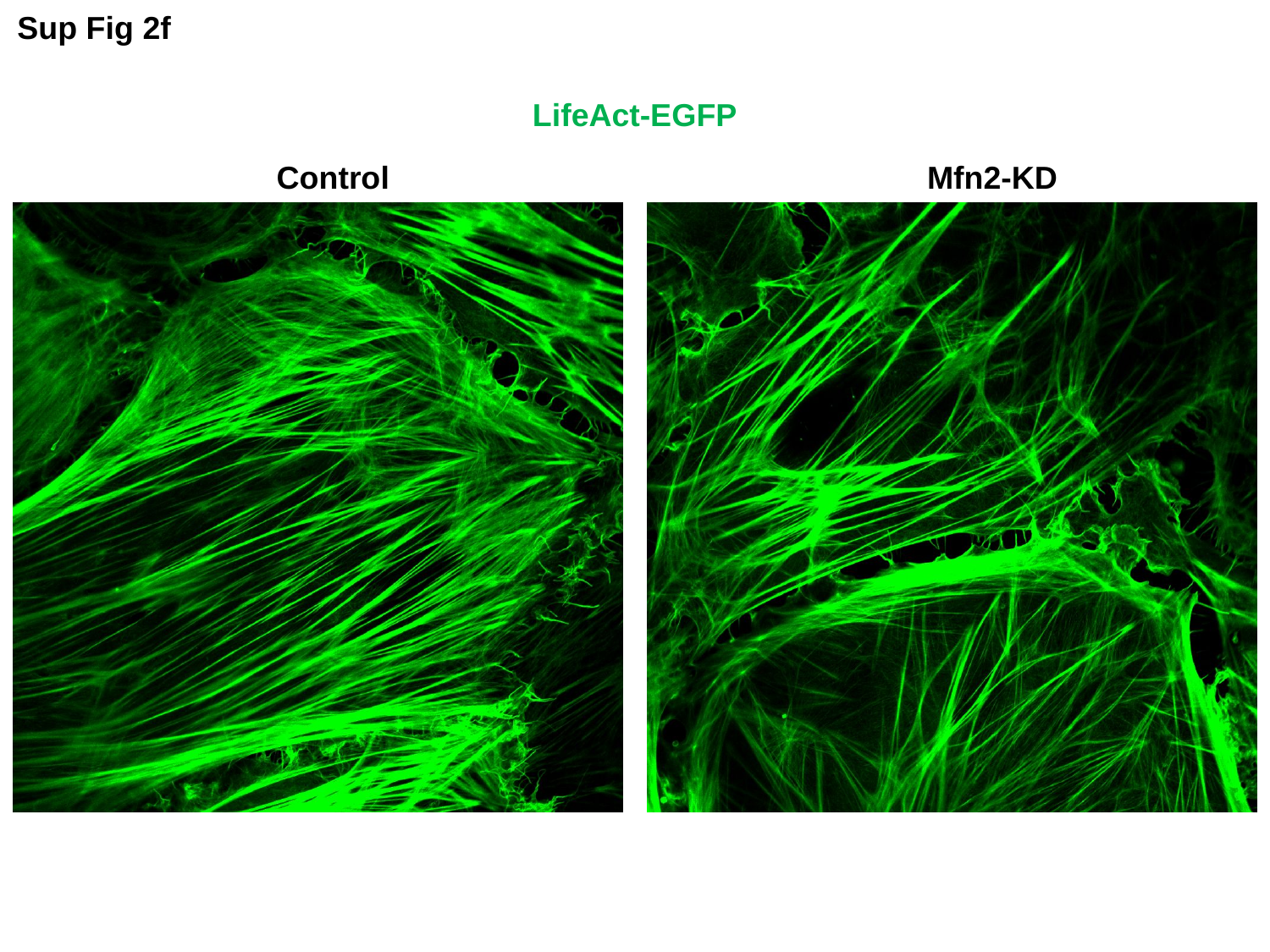

Sup Fig 2f
LifeAct-EGFP
Control
Mfn2-KD

## Slide 6
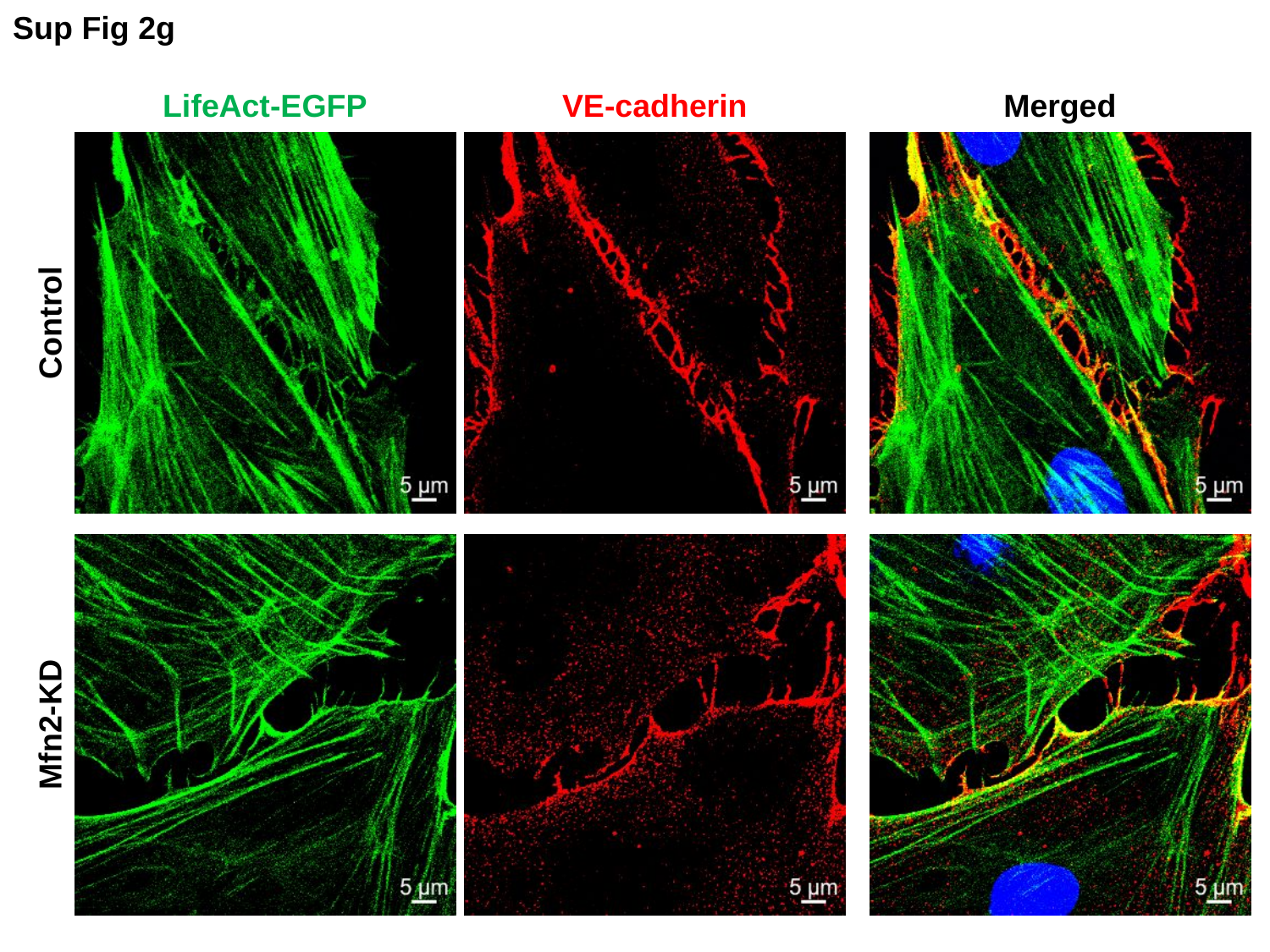

Sup Fig 2g
LifeAct-EGFP
VE-cadherin
Merged
Control
Mfn2-KD

## Slide 7
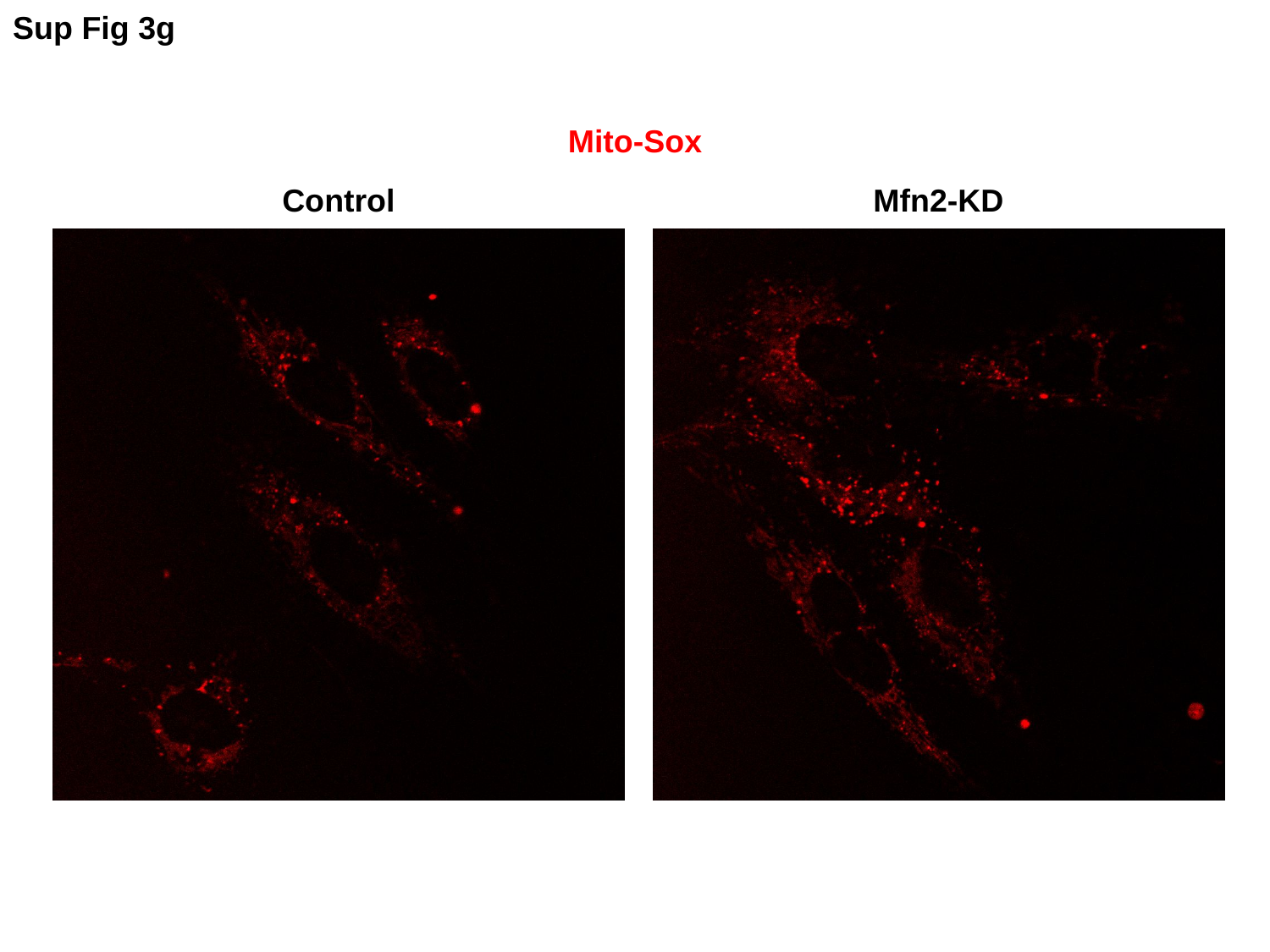

Sup Fig 3g
Mito-Sox
Control
Mfn2-KD

## Slide 8
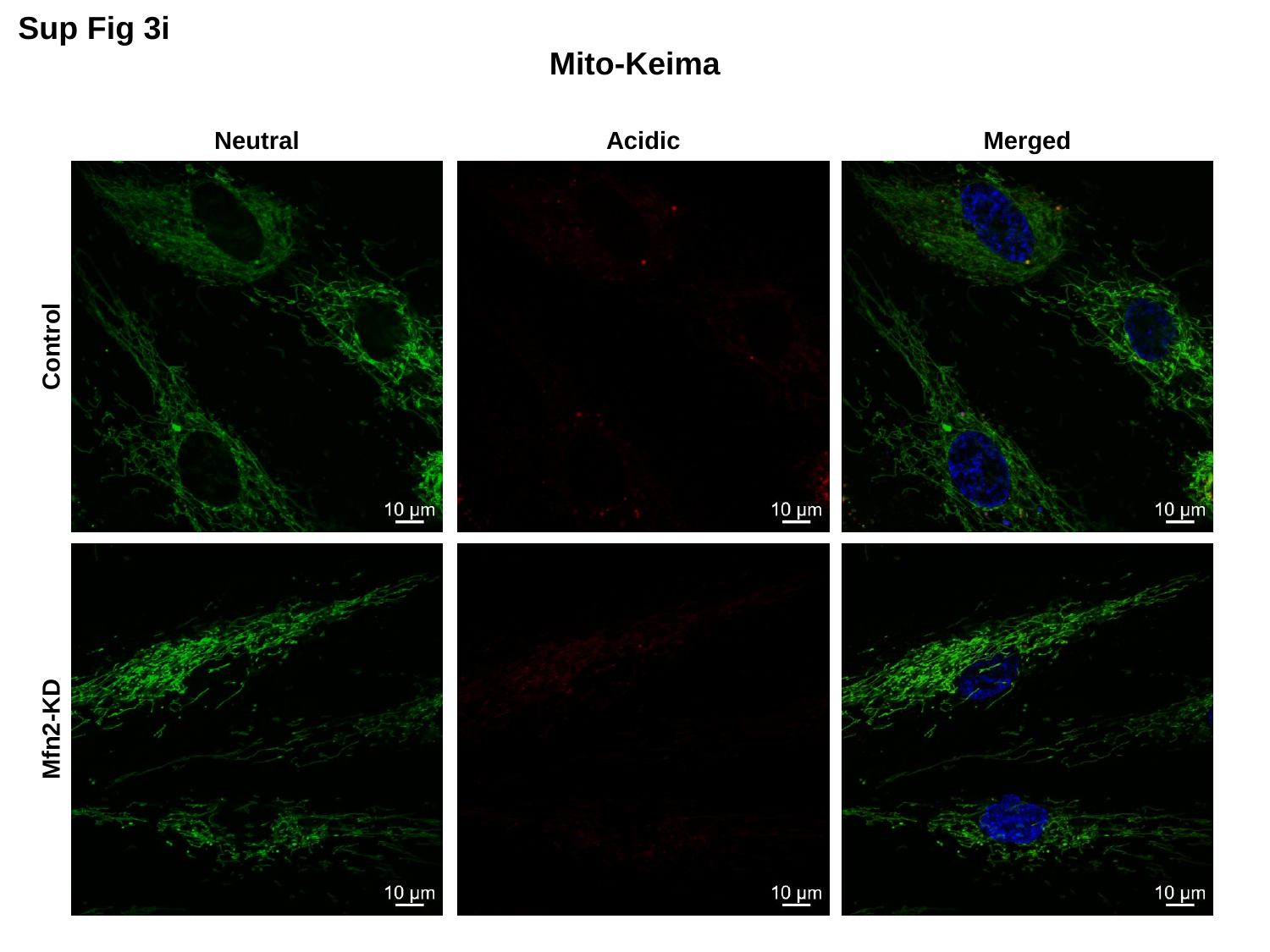

Sup Fig 3i
Mito-Keima
Neutral
Acidic
Merged
Control
Mfn2-KD

## Slide 9
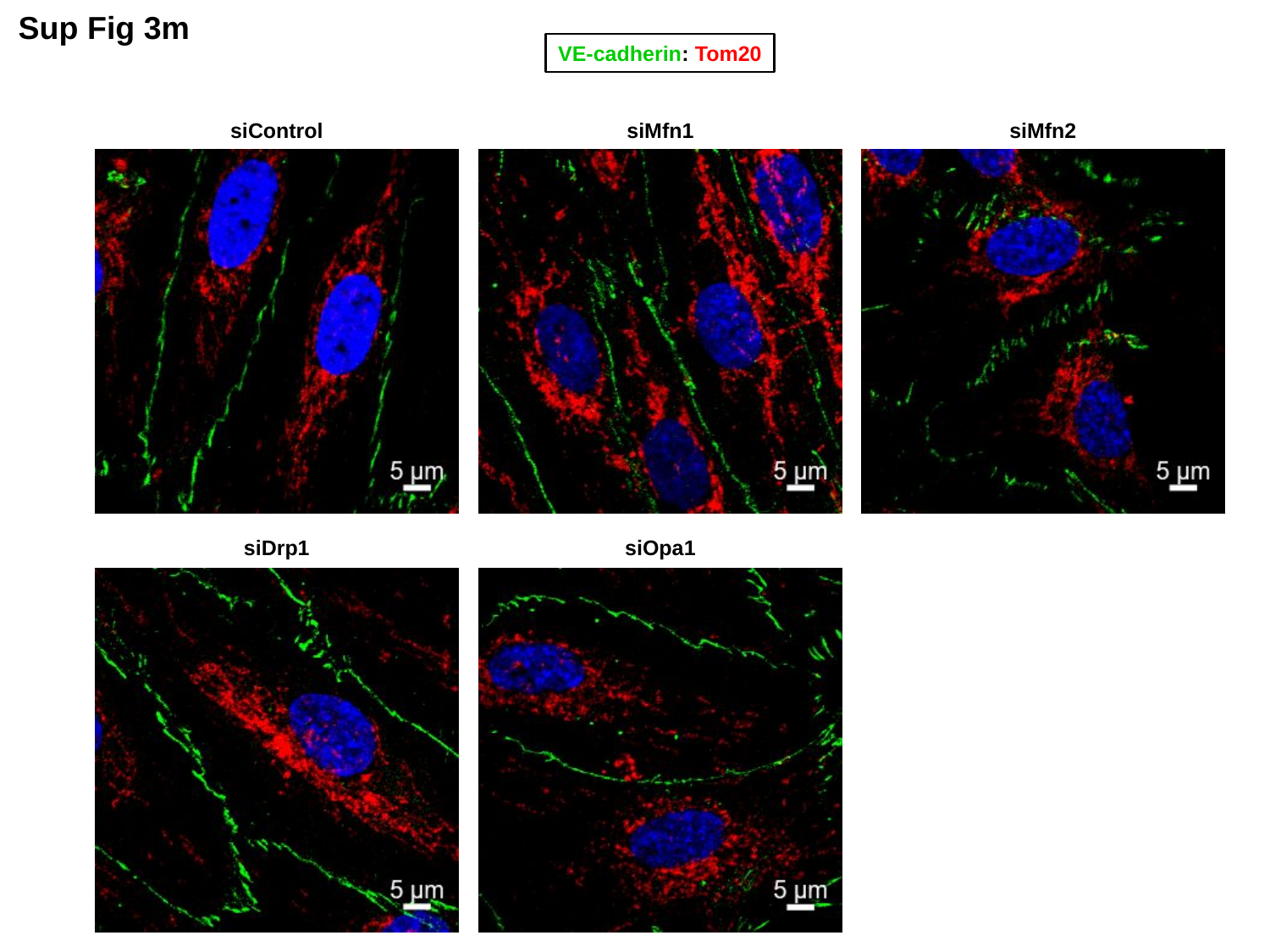

Sup Fig 3m
VE-cadherin: Tom20
siControl
siMfn1
siMfn2
siDrp1
siOpa1

## Slide 10
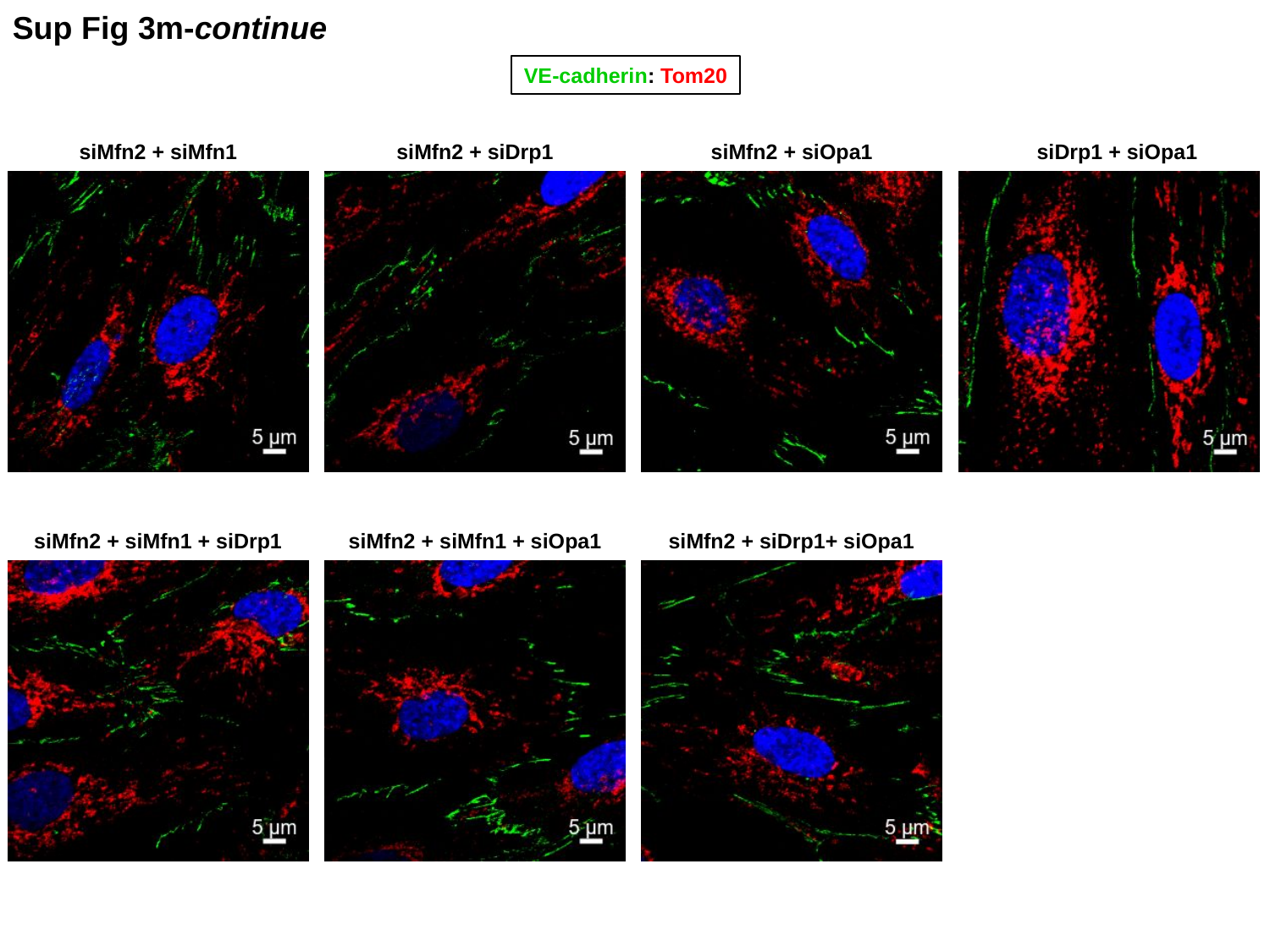

Sup Fig 3m-continue
VE-cadherin: Tom20
siMfn2 + siMfn1
siMfn2 + siDrp1
siMfn2 + siOpa1
siDrp1 + siOpa1
siMfn2 + siMfn1 + siDrp1
siMfn2 + siMfn1 + siOpa1
siMfn2 + siDrp1+ siOpa1

## Slide 11
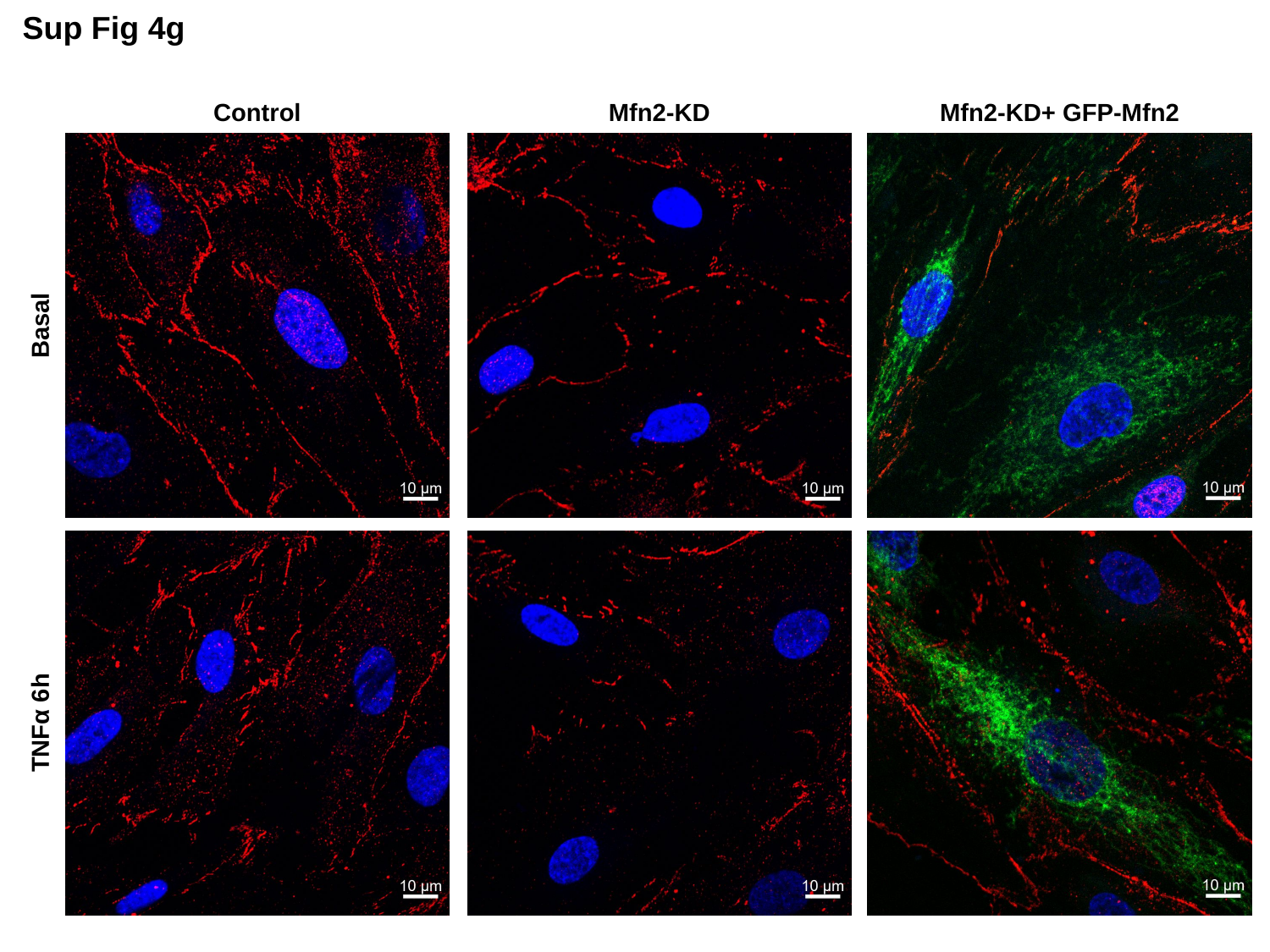

Sup Fig 4g
Control
Mfn2-KD
Mfn2-KD+ GFP-Mfn2
Basal
TNFα 6h

## Slide 12
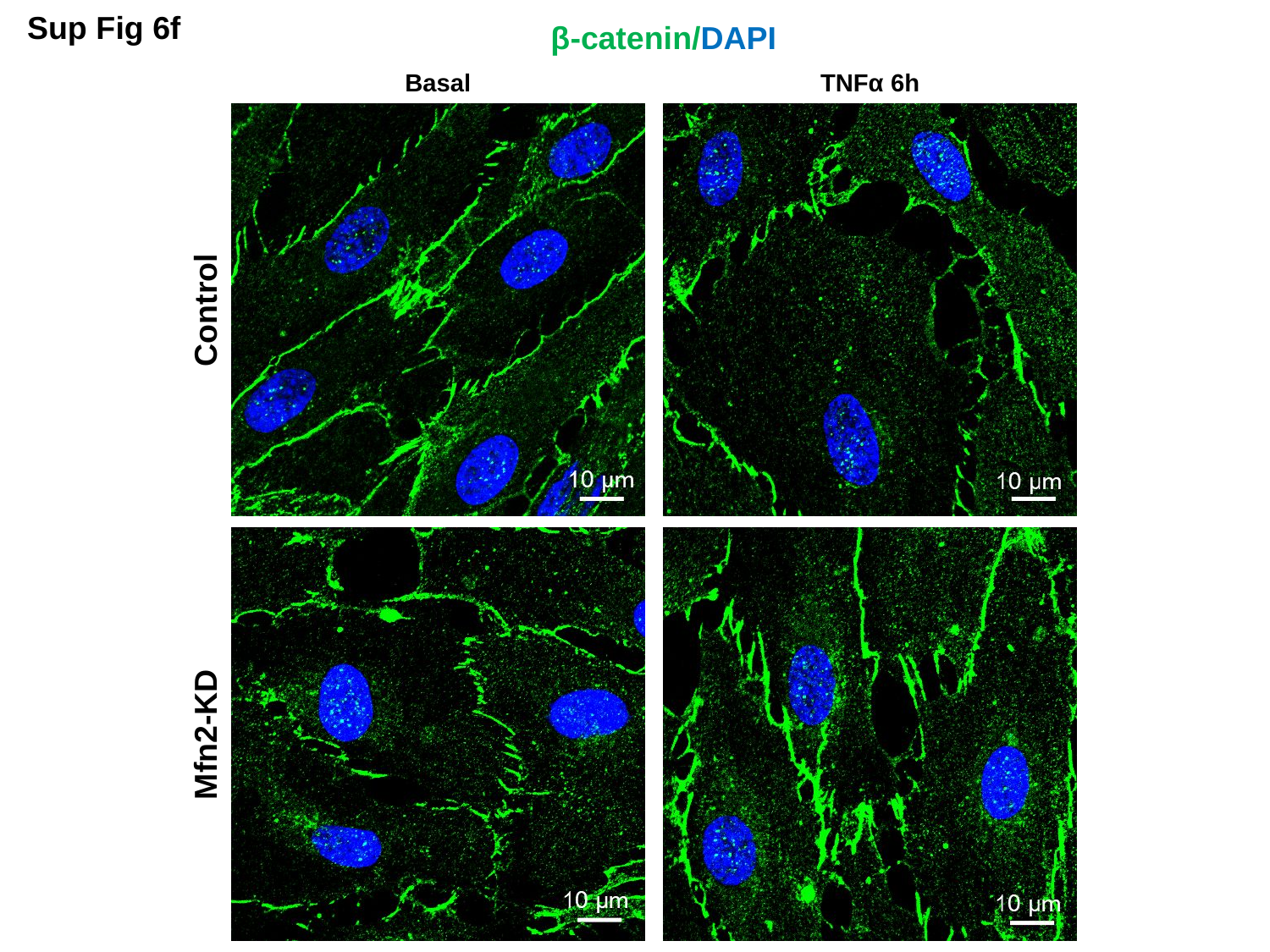

Sup Fig 6f
β-catenin/DAPI
Basal
TNFα 6h
Control
Mfn2-KD
